# Association of Atrial Fibrillation Symptom Burden With Social Determinants of Health

**DOI:** 10.1016/j.jacadv.2025.102302

**Published:** 2025-11-06

**Authors:** Anish S. Shah, Alvaro Alonso, Jaleel Sweis, Sorin Griza, Yining Chen, Miles Barney, Annette Diaz, Bahaa Al-Azzam, Mary R. Ziccardi, Aylin Ornelas-Loredo, Faisal A. Darbar, Amit J. Shah, Emelia J. Benjamin, Dawood Darbar

**Affiliations:** a Department of Medicine, Division of Cardiology, University of Utah, Salt Lake City, Utah, USA; b Department of Medicine, Division of Cardiology, University of Illinois Chicago, Chicago, Illinois, USA; c Department of Medicine, Division of Academic Internal Medicine, University of Illinois Chicago, Chicago, Illinois, USA; d Department of Medicine, Jesse Brown Veterans Administration Medical Center, Chicago, Illinois, USA; e Department of Epidemiology, Rollins School of Public Health, Emory University, Atlanta, Georgia, USA; f Department of Medicine, Advocate Christ Medical Center, Chicago, Illinois, USA; g Department of Medicine, Atlanta VA Medical Center, Decatur, Georgia, USA; h Department of Medicine, Boston Medical Center, Chobanian & Avedisian School of Medicine, Boston University, Boston, Massachusetts, USA; i Department of Epidemiology, School of Public Health, Boston University, Boston, Massachusetts, USA

**Keywords:** atrial fibrillation, patient-reported outcomes, social determinants of health

## Abstract

**Background:**

Social determinants of health (SDoH) may influence the clinical presentation, management, and outcomes related to atrial fibrillation (AF).

**Objectives:**

This study aims to examine the associations of SDoH with trajectories in AF symptom burden in participants undergoing AF treatment.

**Methods:**

The authors designed a prospective, observational cohort study at an academic medical center in a large U.S. metropolitan city serving predominantly low-income and underinsured populations. Participants were those referred with an initial diagnosis of paroxysmal or persistent AF. Adverse SDoH, including NDI (National Deprivation Index), insurance status, language, and marital status, as well as proxies for structural and systemic biases through race, ethnicity, and sex were assessed. Participants received standard care with study-specific follow-up at 1 year. The outcome was scores from the Atrial Fibrillation Effect on Quality-of-Life (AFEQT) questionnaire (higher scores representing better quality of life).

**Results:**

Of 515 participants who had follow-up assessments; 41% were non-Hispanic Black, 40% were non-Hispanic White, and 18% were Hispanic/Latinx. The mean AFEQT score at baseline was 73 (of 100) and at follow-up was 80 (SD: 19). Participants living in higher levels of NDI experienced 6.4 (95% CI: 1.9-11.0) point smaller increase in AFEQT scores compared to those in lower levels of NDI, and non-Hispanic Blacks experienced a 4.6 (95% CI: 1.1-8.0) point smaller increase compared to non-Hispanic Whites. There were 61 admissions for decompensated heart failure, 53 ischemic strokes, and 47 all-cause mortality events.

**Conclusions:**

After adjusting for clinical factors, adverse SDoH associate with blunted improvements in AF symptom burden after 1 year of clinical management.

Atrial fibrillation (AF) is the most common cardiac arrhythmia in adults,^1^^,^^2^ with a lifetime incidence of 1 of every 3 White individuals, and 1 out of every 5 African-American individuals.^3^ The management of AF has traditionally been driven by symptom burden in both treatment initiation and treatment response,^4^ which has led to the development of tools to assess patient-reported outcomes, such as the AFEQT (Atrial Fibrillation Effect on Quality-of-Life) questionnaire.^5^ However critical gaps in knowledge have led to increasing health inequities in the management of AF.^6^ The inequities in AF burden and symptoms,^7^ as well as overall mortality by race and ethnicity,^8^^,^^9^ highlight the importance of the under-representation of minoritized populations in AF clinical trials.^10^ Social determinants of health (SDoH) affect access to care and management in AF, which may drive inequitable outcomes, but few studies have determined their contributions to outcomes.^11^ Evaluating the relationship between SDoH and patient-reported outcomes is the key in understanding how to address health inequities in AF management.

The current guidelines stress the importance of symptoms in driving rhythm-control strategies such as catheter ablation;^12^ however, fewer than 3% of AF studies included patient-reported AF-specific outcome measures,^13^ and of those, the majority focus on changes in episodes of AF as a response to treatment. Contextually, physician estimation of symptom burden is often inaccurate and can lead to underestimation of patient quality of life, which in turn leads to discordant treatment escalation.^14^^,^^15^ In the Outcomes Registry for Better Informed Treatment of Atrial Fibrillation, one of the largest studies to assess AF treatment effects, AFEQT scores were associated with female sex, heart failure, and obstructive pulmonary disease, but psychological, societal, or structural factors were not evaluated.^16^ Other studies are relatively small but provide evidence of the importance of additional risk factors, such as the relationship between AF symptom burden and depressive symptoms or income status.^17^^,^^18^

Our aim was to understand the association of SDoH with symptom burden in patients with AF. We longitudinally evaluated patient-reported AF symptom burden in a multiethnic, multiracial cohort from a large, metropolitan center. We hypothesized that adverse SDoH are associated with increased symptom burden, decreased symptomatic treatment response, and reduced guideline-based treatment (Central Illustration).

**Central Illustration:**
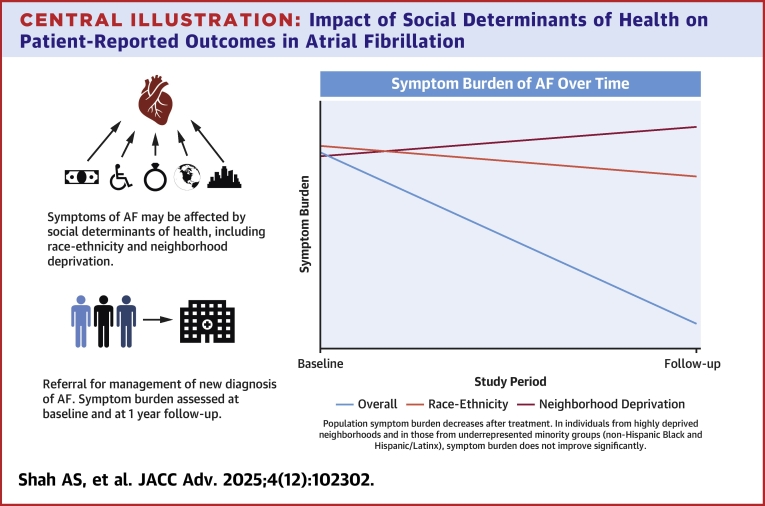
**Impact of Social Determinants of Health on Patient-Reported Outcomes in Atrial Fibrillation** AF = atrial fibrillation.

## Methods

### Study population and protocol overview

**Figure 1:**
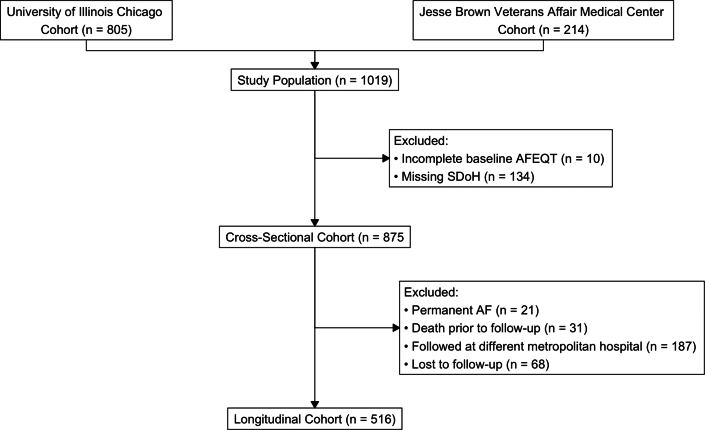
**Flow Diagram of Study Participants** The CONSORT diagram shows the study recruitment and exclusion criteria. Permanent AF was excluded at both time of initial recruitment and if ascertained during the cardiology follow-up. AF = atrial fibrillation; AFEQT = Atrial Fibrillation Effect on Quality-of-Life; SDoH = social determinants of health.

We recruited participants from the UIC (University of Illinois Chicago) and JBVA (Jesse Brown Veterans Affairs) medical centers as part of the UIC multiethnic AF registry from July 1, 2015, to July 1, 2023, as a continuous prospective study focusing on clinical outcomes and genetics in adults with AF.^19^^,^^20^ The enrollment occurred at the time of initial cardiology evaluation and consultation for the diagnosis of AF including both inpatient and outpatient sites, using trained research coordinators interviewing in English or Spanish per patient preference (other languages required use of a virtual translation service). Symptom burden was assessed through the AFEQT questionnaire,^5^ administered at the time of enrollment, and at 12 months of follow-up, if participants were ≥ 18 years of age, had electrocardiogram-confirmed diagnosis of AF prior to enrollment, and were able to complete the AFEQT questionnaire. From the initial enrolled cohort (n = 875), 59% made it to 12 month follow-up (n = 516), with exclusion for those who followed at a different metropolitan hospital (n = 187), were lost to follow-up (n = 68), had mortality events before follow-up (n = 31), or were classified as permanent AF after initial cardiology evaluation (n = 21), as seen in Figure 1. The median (IQR) time from initial diagnosis of AF to cardiology evaluation was 173 (IQR: 9, 783) days. Of the final sample, 59% had initial diagnosis within the 1 year before enrollment and 32% had an initial diagnosis of AF at either UIC or JBVA. The study was approved by the Institutional Review Boards at UIC and JBVA, and all participants provided written informed consent. The study follows the Strengthening the Reporting of Observational Studies in Epidemiology guidelines for observational cohort studies.^21^

### AFEQT scores

The AFEQT questionnaire is a 20-item questionnaire divided into the following 4 conceptual domains: symptoms, daily activities, treatment concerns, and treatment satisfaction.^5^ Both individual domain and global scores are calculated, ranging from 0 to 100, with lower scores representing higher symptom burden and being associated with increased AF severity.^5^ Treatment satisfaction was not consistently administered in our cohort as a majority of participants were newly diagnosed at time of enrollment and not yet on treatment. The AFEQT questionnaire was the chosen instrument as it primarily focuses on patient-reported outcomes, which influence treatment decisions.^4^^,^^12^^,^^16^ The questionnaire can be repeated over time; clinically meaningful changes are defined as a 5-point difference in individual or global domain scores.^22^

### Demographics

Sex, racial identity, and ethnicity were identified through self-report. Race-ethnicity were 1) non-Hispanic Black, 2) Hispanic/Latinx, and 3) non-Hispanic White. No participant identified from an alternative racial or ethnic category.

### SDoH

We collected information on several SDoH potentially related to AF.^6^^,^^7^^,^^11^ Home addresses were deidentified by conversion into geocodes that aligned with census tracts designations by the 2010 and 2020 U.S. Census Bureau. The most recent address was aligned with the appropriate census tract designation. The NDI (National Deprivation Index), a neighborhood-level measure of socioeconomic deprivation and disparity within the United States, was assigned to each participant’s census tract.^23^ The NDI includes census tract–level information on occupation types, crowding, poverty, female-headed households, public assistance requirements, education levels, and employment measurements. The NDI scores were placed within predefined quartiles based on the distribution of these aspatial indices at the national level, from least deprivation to most deprivation. We generated these scores using the *{ndi}* package in *R* (R Core Team).^23^^,^^24^ Higher scores suggest increased deprivation. Insurance type was determined through billing records, and categorized as public (Medicare, Medicaid, or other governmental insurance), private (commercial insurance), or self-pay. Primary spoken language was determined through participant interview and categorized as English, Spanish, or other. Marital status was self-reported and categorized as married/cohabitating, divorced/separated/widowed, or single/never-married.

### Clinical measures

Information on other sociodemographic and clinical factors were determined through standardized questionnaires and chart review. Procedures and medications were evaluated at over a 24-month period, comprising of the 12-month period before enrollment, and the subsequent 12-month period until the follow-up AFEQT questionnaire. The baseline medications were considered those that the participant was prescribed and filled during the 12 months before enrollment, and the follow-up medications were considered those prescribed during the subsequent 12 months until the follow-up visit.

Vital signs were obtained at time of enrollment, which included body mass index (kg/m^2^), systolic and diastolic blood pressure (mm Hg), and heart rate (beats/minute). Coronary artery disease (CAD) was defined as obstructive coronary disease on coronary angiography, abnormal exercise or pharmacological stress testing using either echocardiography or nuclear imaging modalities, prior percutaneous coronary intervention (PCI), or coronary artery bypass grafting (CABG). Heart failure included symptomatic participants requiring diuretic therapy with either preserved or reduced ejection fraction on echocardiogram. Diabetes mellitus was defined by hemoglobin A1c ≥6.5% or by the usage of glucose-lowering therapies with confirmatory diagnosis codes, as described subsequently. Hypertension was defined by the use of antihypertensive agents, systolic blood pressure ≥140 mm Hg, or diastolic blood pressure ≥90 mm Hg with confirmatory diagnosis codes. Stroke and transient ischemic attack, chronic kidney disease, and chronic obstructive pulmonary disease were determined via diagnosis codes.

Participants were seen in clinical settings at UIC or JBVA outpatient clinics during follow-up, with at least 1 cardiology or electrophysiology outpatient visit during the 12-month period after enrollment. Changes in antiarrhythmic drugs (AADs) and antithrombotic medications were extensively reviewed for dose titration, discontinuation of agents, and initiation of alternative agents. AADs were categorized by Vaughn-Williams classification into sodium channel blockers (flecainide, propafenone, and disopyramide), beta blockers (metoprolol, carvedilol, atenolol, and propranolol), potassium channel blockers (amiodarone, dronedarone, sotalol, and dofetilide), and calcium channel blockers (diltiazem and verapamil). We reviewed procedure reports for ablation of atrial arrhythmias, placement of pacemakers and/or implantable cardioverters/defibrillators, and electrical cardioversion events. We defined a rhythm-control strategy as receiving AADs, electrical or pharmacological cardioversion, or AF ablation.

During the follow-up period, we evaluated for major adverse cardiovascular events, including incident diagnosis of CAD with acute coronary syndrome, coronary revascularization through PCI or CABG, incident diagnosis of heart failure at time of hospitalization, incident diagnosis of ischemic stroke, and overall mortality.

### Statistical analysis

Our a priori primary analysis was the relationship between adverse SDoH and the change in global AFEQT score. We performed multivariable linear mixed effects modeling, with all models adjusted for baseline AFEQT score. Our secondary analysis was to assess the association of potential clinical confounders using sequentially adjusted models for sociodemographic factors, cardiovascular risk factors, and major adverse cardiovascular events. The adjustment sets were chosen based on the theoretical causal model associating the clinical risk factors and potential confounders with AF incidence, burden, and outcomes.^12^ Recruitment sites and AF subtypes were random effects, to account for differences in patient populations and the potential differences in symptoms between paroxysmal and persistent AF that may have led to different referral patterns. We secondarily evaluated the association of adverse SDoH with the choice of baseline or follow-up treatment strategy and the switch from a rate-control to rhythm-control strategy. We performed exploratory analyses between adverse SDoH and individual domains of AFEQT scores. For the exploratory analyses, we performed generalized linear mixed effect models with a binomial distribution, adjusting for baseline total AFEQT score. We used sequential multivariable adjustment for traditional factors as previously discussed.

We assessed statistical interaction between individual SDoH with clinical covariates throughsubgroup analyses between paroxysmal and persistent AF subtypes. To assess for the impact on missingness between initial visit and follow-up, we performed testing between cohorts. We compared the distribution of variables between cohorts; used chi-square and Fisher exact tests for categorical variables and independent t-tests or Mann-Whitney U tests for continuous and ordinal data, respectively. The Little test of missing completely at random was used to test the pattern of missingness.

All statistical analyses were performed using *R* (version 4.4.0),^25^ along with supporting packages seen in the supplementary material.^24^^,^^26^, ^27^, ^28^, ^29^ We performed model diagnostics, assessing the linearity of the dependent variables, the homoscedasticity of the residuals, and the normality of the error distributions. Statistical significance was set at α = 0.05 for our primary analyses for 2-sided *P* values, with 95% CIs reported. No multiple comparison adjustment to the α level was made, as individual domain analyses were considered exploratory and sequential adjusted models were considered secondary and contextual.

## Results

### Cohort description

A total of 516 participants had follow-up visits and were available for longitudinal analysis. The mean (SD) age was 64.0 (11.9), 36% were female, 64% were male, 24% were Hispanic/Latinx, 40% were non-Hispanic Black, and 36% were non-Hispanic White. The distributions were similar to those in the full baseline cohort (Supplemental Tables 1 and 2) for SDoH and clinical parameters were seen, with data on missingness reported in Supplemental Table 3.

We present the distribution of the longitudinal cohort characteristics by NDI quartile in Tables 1 and 2. The majority of non-Hispanic Black and Hispanic/Latinx participants were categorized in the highest NDI quartile (71% and 71%, respectively, as row percentages), whereas only 29% as row percentage of White participants were in the highest NDI quartile. The lowest vs highest NDI quartile was primarily insured by public insurance, including Medicare and Medicaid, at similar rates (61% vs 69%). The majority of Spanish-language speakers were in the highest NDI quartile compared to English speakers (81% vs 52%).

**Table 1:** Longitudinal Cohort Description by National Deprivation Index

	Overall (N = 516)	Least Deprivation (n = 69)	Below Average Deprivation (n = 63)	Above Average Deprivation (n = 96)	Most Deprivation (n = 288)
Sociodemographics					
Age, y	64 (58-71)	68 (61-73)	63 (60-70)	63 (58-70)	64 (57-71)
Sex					
Female	185 (36%)	17 (25%)	21 (33%)	23 (24%)	124 (43%)
Male	331 (64%)	52 (75%)	42 (67%)	73 (76%)	164 (57%)
Race and ethnicity					
Non-Hispanic White	184 (36%)	52 (75%)	35 (56%)	44 (46%)	53 (18%)
Hispanic/Latinx	125 (24%)	7 (10%)	14 (22%)	15 (16%)	89 (31%)
Non-Hispanic Black	207 (40%)	10 (14%)	14 (22%)	37 (39%)	146 (51%)
Insurance class					
Private	48 (9.3%)	10 (14%)	9 (14%)	10 (10%)	19 (6.6%)
Public	353 (68%)	42 (61%)	41 (65%)	72 (75%)	198 (69%)
Self-pay	115 (22%)	17 (25%)	13 (21%)	14 (15%)	71 (25%)
Language group					
English	445 (86%)	67 (97%)	57 (90%)	88 (92%)	233 (81%)
Other primary language	4 (0.8%)	0 (0%)	2 (3.2%)	1 (1.0%)	1 (0.3%)
Spanish	67 (13%)	2 (2.9%)	4 (6.3%)	7 (7.3%)	54 (19%)
Marital status					
Married or cohabitating	216 (42%)	37 (54%)	28 (44%)	41 (43%)	110 (38%)
Divorced, separated, or widowed	109 (21%)	13 (19%)	17 (27%)	19 (20%)	60 (21%)
Single or never-married	191 (37%)	19 (28%)	18 (29%)	36 (38%)	118 (41%)
Clinical covariates					
Coronary artery disease	102 (20%)	9 (13%)	13 (22%)	22 (24%)	58 (20%)
Congestive heart failure	212 (42%)	21 (30%)	21 (35%)	37 (40%)	133 (47%)
Stroke/TIA	90 (18%)	9 (13%)	11 (18%)	16 (17%)	54 (19%)
Diabetes mellitus	221 (44%)	20 (29%)	25 (42%)	36 (39%)	140 (49%)
Hypertension	392 (76%)	46 (67%)	45 (71%)	70 (73%)	231 (80%)
Chronic kidney disease	139 (28%)	13 (19%)	21 (35%)	16 (17%)	89 (31%)
COPD	126 (25%)	9 (13%)	12 (20%)	22 (24%)	83 (29%)
CHA2DS2VASc score	3 (2-5)	3 (2-4)	3 (2-4)	3 (1-4)	4 (2-5)
Type of AF					
Paroxysmal	411 (80%)	52 (75%)	52 (83%)	69 (72%)	238 (83%)
Persistent	105 (20%)	17 (25%)	11 (17%)	27 (28%)	50 (17%)
Previous cardiac procedures					
Pacemaker or defibrillator implant	24 (4.7%)	3 (4.3%)	2 (3.2%)	4 (4.2%)	15 (5.2%)
Electrical direct-current cardioversion	60 (12%)	9 (13%)	11 (17%)	7 (7.3%)	33 (11%)
Catheter ablation	48 (9.3%)	8 (12%)	1 (1.6%)	9 (9.4%)	30 (10%)
Baseline treatment strategy					
Anticoagulant agents	383 (74%)	49 (71%)	43 (68%)	76 (79%)	215 (75%)
Rate-control medications	410 (79%)	51 (74%)	52 (83%)	68 (71%)	239 (83%)
Rhythm-control medications	161 (31%)	24 (35%)	21 (33%)	38 (40%)	78 (27%)
Rhythm-control strategy	188 (36%)	29 (42%)	21 (33%)	42 (44%)	96 (33%)

Values are median (Q1-Q3) or n (%).

COPD = chronic obstructive pulmonary disease; TIA = transient ischemic attack.

**Table 2:** Longitudinal Cohort Treatment Characteristics by National Deprivation Index

	Overall (N = 516)	Least Deprivation (n = 69)	Below Average Deprivation (n = 63)	Above Average Deprivation (n = 96)	Most Deprivation (n = 288)
Follow-up medications					
Antiplatelet agents	212 (41%)	22 (32%)	24 (38%)	31 (32%)	135 (47%)
Anticoagulant agents	353 (68%)	38 (55%)	43 (68%)	56 (58%)	216 (75%)
Sodium channel blockers	71 (16%)	8 (14%)	13 (25%)	15 (20%)	35 (14%)
Beta blockers	346 (67%)	37 (54%)	45 (71%)	62 (65%)	202 (70%)
Potassium channel blockers	134 (26%)	13 (19%)	20 (32%)	21 (22%)	80 (28%)
Calcium channel blockers	56 (11%)	5 (7.2%)	8 (13%)	11 (11%)	32 (11%)
Follow-up treatment strategy					
Rate-control medications	360 (70%)	39 (57%)	46 (73%)	66 (69%)	209 (73%)
Rhythm-control medications	179 (40%)	18 (32%)	29 (54%)	31 (39%)	101 (39%)
Rhythm-control strategy	260 (55%)	34 (52%)	36 (63%)	46 (55%)	144 (53%)
Catheter ablation	68 (13%)	9 (13%)	8 (13%)	15 (16%)	36 (13%)
Electrical direct-current cardioversion	54 (10%)	6 (8.7%)	7 (11%)	10 (10%)	31 (11%)
Baseline AFEQT scores					
Overall	79 (57-92)	81 (65-94)	73 (48-92)	78 (55-92)	79 (60-92)
Symptoms	88 (67-100)	88 (63-100)	92 (63-100)	83 (50-100)	92 (67-100)
Daily activities	73 (44-96)	83 (48-100)	73 (38-95)	72 (42-95)	71 (46-95)
Treatment concerns	83 (61-100)	82 (64-100)	86 (50-100)	85 (58-100)	83 (64-100)
Changes in AFEQT scores					
Overall (change)	5 (−5 to 20)	8 (−1 to 19)	6 (−2 to 23)	7 (−5 to 21)	3 (−7 to 20)
Symptoms (change)	0 (−4 to 17)	8 (0-21)	0 (0-17)	4 (0-25)	0 (−4 to 17)
Daily activities (change)	2 (−8 to 25)	0 (−4 to 29)	7 (−2 to 35)	2 (−13 to 24)	2 (−10 to 23)
Treatment concerns (change)	0 (−6 to 22)	6 (−3 to 22)	0 (0-20)	3 (0-22)	0 (−8 to 22)
Major adverse cardiovascular events					
Acute coronary syndrome	56 (13%)	7 (13%)	6 (11%)	8 (9.8%)	35 (14%)
Coronary revascularization	18 (3.5%)	2 (2.9%)	1 (1.6%)	7 (7.3%)	8 (2.8%)
Acute decompensated heart failure	61 (14%)	9 (15%)	8 (15%)	11 (13%)	33 (13%)
Acute ischemic stroke	53 (13%)	3 (5.8%)	6 (12%)	10 (13%)	34 (14%)
All-cause mortality	47 (9.1%)	2 (2.9%)	6 (9.5%)	9 (9.4%)	30 (10%)

Values are median (Q1-Q3) or n (%).

AFEQT = Atrial Fibrillation Effect on Quality-of-Life.

The distribution of the longitudinal cohort characteristics by race and ethnicity are seen in Supplemental Tables 4 and 5. Insurance types were also similar across race and ethnic groups. There was a greater number of women that identified as non-Hispanic Black participants than as White participants (45% vs 21%).

A total of 875 participants had baseline data and were available for initial cross-sectional analysis. The mean (SD) age was 63.7 (12.5). 36% were female, 56% were male, 18% were Hispanic/Latinx, 41% were non-Hispanic Black, and 40% were non-Hispanic White. The highest quartile of NDI, representing the most deprivation, comprised 56% of the cohort. For insurance coverage, 68% had public insurance and 21% were self-pay. Spanish was the primary language for 10% of the cohort. Relationship status was obtained for participants, and at time of enrollment 39% were never married and 21% were divorced or separated. Additional clinical characteristics are shown in Supplemental Table 2.

### Clinical outcomes

The median (IQR) of CHA2DS2VASc scores was 3 [3]. The prevalence of CAD was 20%, systolic or diastolic heart failure was 41%, stroke or transient ischemic attack was 17%, diabetes mellitus was 43%, and hypertension was 76%. During the follow-up period, there were 56 acute coronary syndrome events, 18 coronary interventions through either CABG or PCI, 61 acute decompensated heart failure hospitalizations, and 53 acute ischemic strokes. From the initial visit before follow-up, there were 31 mortality events, with an additional 47 that occurred after the final follow-up appointment (Table 2, Supplemental Table 5).

### Association of SDoH with AFEQT

**Figure 2:**
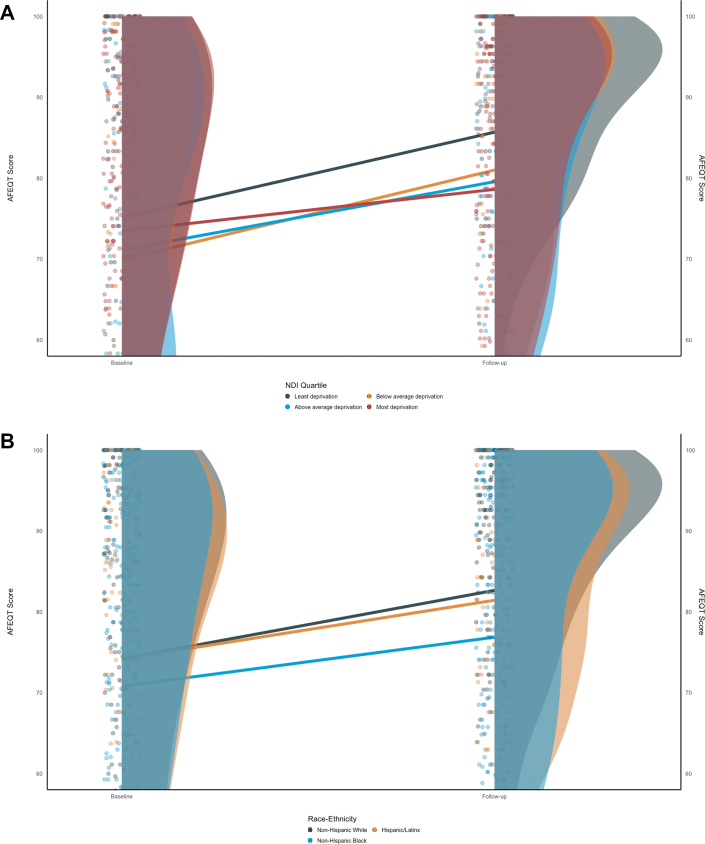
**Distribution and Change in AFEQT Scores Across Social Determinants of Health** (A) shows the change in AFEQT over the study period based on underlying NDI quartile. There is an increase in AFEQT scores most strongly seen in the lowest deprivation group. (B) shows the change in AFEQT over the study period based on underlying Race-Ethnicity. There is an increase in AFEQT scores most strongly seen in the Non-Hispanic White group. The initial and follow-up distributions are composed of only those participants in the longitudinal cohort (n = 516). Ridgeline plots are drawn to show the distribution of based on groups of NDI quartile or race-ethnicity. The density of individuals is visualized as a scatter plot adjacent to the ridgelines. The regression lines show the average change in AFEQT from baseline to follow-up time point in models adjusted for baseline AFEQT scores. NDI = National Deprivation Index; other abbreviations as in Figure 1.

The median [IQR) AFEQT scores of the full baseline cohort were 78 (35), and 86 (28) at follow-up (Supplemental Tables 1 and 2). When restricted to only those in the longitudinal cohort, the initial AFEQT score was 79 (35). The AFEQT scores at baseline and follow-up of the longitudinal cohort are shown in Figure 2.

In Table 3, we evaluated the association of SDoH on the change in AFEQT scores at follow-up, accounting for the baseline AFEQT score (Model 1). We observed that higher NDI quartiles had blunted improvements in AFEQT score (higher AFEQT scores represent higher quality of life), with the highest deprivation group having a −6.4 (95% CI: −11.0 to −1.9) lower increase in AFEQT total score at follow-up compared to the lowest deprivation group. Non-Hispanic Black participants had a −4.5 (95% CI: −8.0 to −1.1) lower increase in the AFEQT total score at follow-up compared to non-Hispanic White participants. Continuous NDI had a similar association between changes in AFEQT scores at follow-up, but was found to be nonlinear, justifying the usage of quartiles.

**Table 3:** AFEQT Global Score and Social Determinants of Health With Traditional Risk Factors

Change in Total AFEQT Score^e^	NDI Quartile (Least Deprivation)^a^			Race and Ethnicity (Non-Hispanic White)^b^		Sex (Female)	Insurance (Private)^c^		Language (English)^d^		Marital Status (Married or Cohabitating)	
	Below Average Deprivation	Above Average Deprivation	Most Deprivation	Hispanic/Latinx	Non-Hispanic Black	Male	Public	Self-Pay	Other Primary Language	Spanish	Divorced, Separated, or Widowed	Single or Never-Married
Model 1 = adjustment for baseline AFEQT^f^	−2.7 (−8.7, 3.2)	−4.6 (−10.0, 0.8)	−6.4 (−11.0, −1.9)	−1.2 (−5.2, 2.7)	−4.5 (−8.0, −1.1)	1.2 (−1.9, 4.4)	−0.6 (−5.9, 4.6)	−1.7 (−7.6, 4.2)	3.2 (−14.0, 20.3)	3.1 (−1.4, 7.6)	−2.3 (−6.3, 1.7)	−0.1 (−3.5, 3.3)
Model 2 = Model 1 + age + BMI + sociodemographic factors^g^	−2.4 (−8.4, 3.5)	−4.2 (−9.6, 1.2)	−6.2 (−10.7, −1.6)	−1.2 (−5.1, 2.8)	−4.3 (−7.8, −0.9)	1.4 (−1.7, 4.6)	−1.5 (−6.8, 3.9)	−2.7 (−8.7, 3.2)	3.5 (−13.7, 20.7)	2.5 (−2.0, 7.1)	−3.2 (−7.3, 0.9)	0.7 (−2.7, 4.2)
Model 3 = Model 2 + cardiovascular risk factors^h^	−2.3 (−8.4, 3.8)	−4.1 (−9.6, 1.4)	−6.2 (−10.9, −1.5)	−1.6 (−5.7, 2.5)	−4.6 (−8.1, −1.0)	1.5 (−1.7, 4.7)	−1.6 (−7.0, 3.8)	−2.8 (−8.9, 3.3)	3.8 (−13.6, 21.2)	2.6 (−2.1, 7.3)	−3.2 (−7.4, 1.0)	1.0 (−2.5, 4.5)
Model 4 = Model 3 + major cardiovascular adverse events^i^	−2.8 (−8.9, 3.2)	−4.5 (−10.0, 0.9)	−6.2 (−10.9, −1.6)	−1.1 (−5.2, 2.9)	−4.1 (−7.6, −0.5)	0.8 (−2.5, 4.0)	−1.1 (−6.5, 4.3)	−2.0 (−8.1, 4.1)	4.2 (−13.1, 21.5)	2.9 (−1.8, 7.6)	−2.5 (−6.7, 1.7)	1.9 (−1.7, 5.4)
Model 5 = Model 4 + treatment strategy	−2.8 (−8.9, 3.3)	−4.5 (−10.0, 0.9)	−6.3 (−10.9, −1.6)	−1.1 (−5.2, 2.9)	−4.1 (−7.6, −0.5)	0.8 (−2.5, 4.0)	−1.1 (−6.5, 4.3)	−2.0 (−8.1, 4.1)	4.2 (−13.2, 21.5)	2.9 (−1.8, 7.6)	−2.5 (−6.7, 1.7)	1.9 (−1.7, 5.4)

BMI = body mass index, calculated as weight in kilograms divided by height in meters squared; NDI = National Deprivation Index; other abbreviations as in Table 2.

a The NDI quartiles were determined based on comparison of the NDI at the selected census track compared to the national average. The referent group was the bottom quartile of NDI, described as tracks with the least deprivation (n = 69). The below average (n = 63), above average (n = 96), and most deprivation (n = 288) quartiles were considered as ordinal categories for comparison.

b These categories were determined by self-report. Non-Hispanic Black (n = 207) and Hispanic/Latinx (n = 125) were compared with Non-Hispanic White (n = 184) participants as the reference for all models.

c Public insurance (n = 353) and self-pay (n = 115) insurance were compared against private (n = 48) insurance, which was the reference group for all models.

d Language was confirmed via patient interview. Spanish (n = 67) was selected if a translator was required for the interview, or was categorized as “other primary language” (n = 4). The referent language for models was English (n = 445).

e Linear mixed effects models for the continuous change in AFEQT score, with random effects for site and subtype of AF. Beta estimates (95% CI) are presented.

f As the outcome was defined as change in AFEQT over study period, all models adjusted for baseline AFEQT scores.

g Sociodemographic factors included age, race and ethnicity, and sex, when not included as the primary exposure).

h Cardiovascular risk factors included obesity, smoking, hypertension, diabetes, kidney disease, and hyperlipidemia.

i The prevalent major cardiovascular adverse events included coronary artery disease, cerebrovascular accident (stroke or TIA), congestive heart failure, or peripheral vascular disease.

With sequential adjustment, we observed that both the highest deprivation quartile, compared to the lowest, and non-Hispanic Black participants, compared to non-Hispanic White participants, had a −6.3 (95% CI: −10.9 to −1.6) and −4.1 (95% CI: −7.6 to −0.5) lower increase in the AFEQT total score, respectively (Model 5), regardless of the treatment strategy (rhythm-control, cardioversion, or ablation).

In our exploratory analyses, non-Hispanic Black participants had a −7.1 (95% CI: −11.0 to −3.1) and Hispanic/Latinx participants had a −5.1 (95% CI: −9.6 to −0.6) lower increase in the AFEQT treatment domain score, as compared to non-Hispanic White participants. Spanish speaking participants had an 8.3 (95% CI: 2.2-14.5) higher increase in the AFEQT activity domain score at follow-up compared to English speakers. Our reported findings were similar with multivariable adjustment (Supplemental Tables 6 to 8).

The AFEQT scores at baseline had no significant association with SDoH, with and without multivariable adjustment, except for with sex. Men had a 4.1 (95% CI: −0.08 to 8.2) higher AFEQT total score at baseline compared to women (adjusted: 4.6 [95% CI: 0.4-8.7]). The exploratory individual AFEQT domain scores were similar to the overall composite AFEQT scores. In the symptoms subscale, men had a 6.70 (95% CI: 1.91-11.50) higher score then women. The estimates were similar to adjusted models (Supplemental Table 9). The baseline cohort and longitudinal cohort had similar patterns of baseline and missing data (Little test, *P* > 0.05) for the chosen predictor and response variables, without strong evidence against being missing at random.

### Rate and rhythm control strategies

In the baseline cohort, 29% were allocated to a rhythm-control strategy (using AADs, ablation, and/or cardioversion approaches), similar to the initial treatment strategy in the longitudinal cohort (Supplemental Table 10). Over the follow-up period, 50% of all participants were on a rhythm-control strategy. We observed no significant associations with baseline treatment strategy and SDoH. Over the follow-up period, non-Hispanic Black participants, compared to non-Hispanic White participants, were less likely to be treated with a rhythm control strategy (OR: 0.6; 95% CI: 0.4-0.9). There were no significant associations for the odds of switching from a rate-control to rhythm-control strategy. There was no difference in referral to ablation by race-ethnicity or NDI quartile at baseline or follow-up (*P* = 0.3 and *P* = 0.6, respectively). Further model details are shown in Supplemental Table 11.

## Discussion

In this prospective, real-world cohort of patients with AF, the specific SDoH of NDI and racism (through proxy of race and ethnicity) were significantly associated with relative worsening in AFEQT scores across individual and global domains. The blunted responses in AFEQT scores were most prominent for higher deprivation in the NDI, compared to less deprivation and race and ethnicity, with non-Hispanic Black participants with AF compared to non-Hispanic White participants, having a relative lower quality-of-life improvement with treatment. The findings were consistent when adjusting for other sociodemographic and clinical risk factors. In exploratory analyses among the individual domains, there was an increase in the AFEQT activity domain in Spanish speakers compared to English speakers. During the follow-up period, there was a lower likelihood of receiving a rhythm-control strategy in non-Hispanic Black compared to non-Hispanic White participants.

The recognition of the importance of SDoH in AF management has been rising,^11^ and there are now limited studies that have evaluated the relationship with patient-reported outcomes of quality of life but even fewer that have looked at AFEQT scores over time. Studies have mostly focused on the effect of rhythm control strategies on the quality of life, primarily with ablation through pulmonary vein isolation, with usual AFEQT score improvements of 25 to 35 points.^30^^,^^31^ In the Systematic Geriatric Elements in Atrial Fibrillation study,^32^ one of the largest studies evaluating AFEQT changes over time, worse AFEQT scores were associated with higher reports of anxiety and depression, but this was limited to a predominantly White population of European ancestry over the age of 75 years. Gleason et al (2019) demonstrated a relationship of sex, age, and income status with AFEQT scores;^33^ however, the association was generally weak (<5 points change on the AFEQT score), which is not considered a clinically meaningful difference,^22^ and the sample was similarly older (>70 years) and predominantly White.

Other studies have reported some associations with psychosocial stress, income levels, and AFEQT scores; however, these were generally cross-sectional and smaller in size.^34^, ^35^, ^36^ The NDI and, by proxy, race and ethnicity are potential surrogates for chronic psychosocial stress, which is directly linked to abnormalities in cardiac function and changes in sympathovagal balance and systemic inflammation.^37^, ^38^, ^39^, ^40^, ^41^ The fact that quality of life can be improved or influenced through indirect pathways, such as cognitive behavioral therapy,^42^ suggests that neurocardiac disturbances in AF at least partially drive symptom burden. The importance of alternative stress pathways in AF arrhythmogenesis is gaining traction, particularly as the role of fibrosis and scar do not fully explain AF burden.^43^^,^^44^

Our study suggests that even with consideration for ablation and heart failure status, traditional cardiovascular factors did not fully explain the association of SDoH with quality of life. Provider awareness of the connection between inequities on symptom perception, clinical AF recognition, and health care access is crucial, particularly as worse quality of life was seen in non-Hispanic Black and Hispanic/Latinx participants.^45^ The reduced likelihood of receiving a rhythm-control strategy in non-Hispanic Black participants needs to be further explored, for example, there may be concerns about side effects of AADs and ablation that are confounded by differences in health literacy and physician–patient relationships, or the differences in self-advocacy or lack of trust in medical care.^46^ Similarly, a physician–patient relationship may also allow improved perception of symptoms and treatment response, such as the increased activity levels seen in Spanish speakers, potentially related to cultural norms and language barriers. Consideration of how symptoms are elicited is also crucial as physician estimation is often inaccurate,^14^^,^^15^ and there are racial differences in AF symptom reporting.^6^^,^^20^^,^^47^^,^^48^

Future studies should focus on both symptom burden and underlying atrial myopathy. We do not know if earlier ablative therapy coupled with adjunctive therapies, such as stress reduction techniques, improve symptom burden and prevent or reverse atrial remodeling. We also do not know if the association of SDoH on symptom burden are modifiable or open to intervention. Finally, we recommend a more granular evaluation of social determinants, including household factors, education, job type and status, and income levels, which may provide insight into specific modifiable pathways and personalization of treatment strategies.

### Study Limitations

The study recruited from 2 institutions in a metropolitan city, which is likely biased on the patient populations primarily served, with frequent turnover and transfers of care. We submit that this is more representative of a real-world population in an underserved area, but it is a single city in the United States and does not account for selection and attrition biases. Mortality events also led to attrition and may contribute to the lack of follow-up, which appeared to be not completely at random in our analyses, with a bias toward missing mortality events in the non-Hispanic White population. We were not able to collect information on major bleeding events in our population, nor were we able to adjudicate the stroke etiology (eg, cardioembolic); however, we were able to differentiate between new-onset of most major adverse cardiovascular events. We were not able to obtain complete data on individual education level nor income level and relied on NDI as a surrogate for these factors. Changes in SDoH factors were not able to be captured in our study, limiting understanding of dynamic changes in SDoH and their impact on symptom burden. Clinical markers of mental health, such as depressive or anxiety disorders, may also confound the relationship with symptom burden, but this data were not consistently available within the multi-institution data.

## Conclusions

In this moderately large, prospectively-enrolled pragmatic cohort of participants with AF, we observed that SDoH, particularly neighborhood-based deprivation scores and race and ethnicity, were significant and had a meaningful association on the quality of life over the first year of follow-up.^22^ Our findings were robust to adjustment for traditional cardiovascular risk factors, treatment strategy, recruitment bias, and type of AF. Our findings provide strong evidence for the importance of social determinants and patient-reported outcomes in understanding AF.

Perspectives **COMPETENCY IN SYSTEMS-BASED PRACTICE:** The management of AF requires additional consideration of how symptom burden and treatment response are assessed when considering both rate and rhythm control strategies.

**TRANSLATIONAL OUTLOOK:** The association of SDoH with AF symptom burden suggests the need to better understand psychosocial stressors and their impact on therapeutic patient alliances and patient-reported outcomes. Further studies are needed to understand how symptom burden may be modified in a more holistic manner.

## Funding support and author disclosures

This work was supported by grants F32HL154707 (Dr A.S. Shah), T32HL139439 (Dr Shah), R01HL150586 (Dr D. Darbar), R01HL148444 (Dr D. Darbar), R01HL092577 (Dr Benjamin) from the 10.13039/100000050National Heart, Lung and Blood Institute, and by grants, 837198, 18SFRN34110082 (Dr Benjamin) by the 10.13039/100000968American Heart Association. The authors have reported that they have no relationships relevant to the contents of this paper to disclose.
