# Supplementary material for: Association of Atrial Fibrillation Symptom Burden With Social Determinants of Health
Source: JACC Adv. 2025 Nov 6;4(12):102302. doi: 10.1016/j.jacadv.2025.102302 (PMC12793848; doi:10.1016/j.jacadv.2025.102302)
Supplement: Supplemental Tables 1-11 [file mmc1.pdf]

SUPPLEMENTAL MATERIALS

The following supplemental tables are contained in the following order below:

- Table S1
- Table S2
- Table S3
- Table S4
- Table S5
- Table S6
- Table S7
- Table S8
- Table S9
- Table S10
- Table S11

Table S1: ***Longitudinal Cohort Description by AFEQT Score at Follow-up***

	**Overall** N = 516[a]	**Q1 (5 - 58)** N = 132[a]	**Q2 (58 - 78)** N = 121[a]	**Q3 (78 - 93)** N = 141[a]	**Q4 (93 - 100)** N = 122[a]
Sociodemographics					
Age (years)	64 (58, 71)	62 (56, 69)	63 (57, 70)	65 (60, 73)	67 (59, 74)
Sex					
Female	185 (36%)	56 (42%)	43 (36%)	44 (31%)	42 (34%)
Male	331 (64%)	76 (58%)	78 (64%)	97 (69%)	80 (66%)
Race & Ethnicity					
Non-Hispanic White	184 (36%)	45 (34%)	41 (34%)	52 (37%)	46 (38%)
Hispanic/Latinx	125 (24%)	29 (22%)	30 (25%)	39 (28%)	27 (22%)
Non-Hispanic Black	207 (40%)	58 (44%)	50 (41%)	50 (35%)	49 (40%)
Neighborhood deprivation index by quartile					
Least deprivation	69 (13%)	14 (11%)	17 (14%)	19 (13%)	19 (16%)
Below average deprivation	63 (12%)	21 (16%)	13 (11%)	14 (9.9%)	15 (12%)
Above average deprivation	96 (19%)	30 (23%)	19 (16%)	27 (19%)	20 (16%)
Most deprivation	288 (56%)	67 (51%)	72 (60%)	81 (57%)	68 (56%)
Insurance Class					
Private	48 (9.3%)	11 (8.3%)	14 (12%)	9 (6.4%)	14 (11%)
Public	353 (68%)	91 (69%)	82 (68%)	98 (70%)	82 (67%)
Self-Pay	115 (22%)	30 (23%)	25 (21%)	34 (24%)	26 (21%)
Language Group					
English	445 (86%)	117 (89%)	103 (85%)	121 (86%)	104 (85%)
Other primary language	4 (0.8%)	0 (0%)	2 (1.7%)	1 (0.7%)	1 (0.8%)
Spanish	67 (13%)	15 (11%)	16 (13%)	19 (13%)	17 (14%)
Marital Status					
Married or cohabitating	216 (42%)	53 (40%)	48 (40%)	68 (48%)	47 (39%)
Divorced, separated, or widowed	109 (21%)	24 (18%)	27 (22%)	32 (23%)	26 (21%)
Single or never-married	191 (37%)	55 (42%)	46 (38%)	41 (29%)	49 (40%)
Vital Signs					
Body mass index (kg/m^2)	30 (26, 35)	29 (26, 35)	31 (25, 39)	30 (28, 34)	29 (27, 35)
Systolic blood pressure (mmhg)	131 (119, 141)	127 (117, 134)	131 (119, 141)	134 (125, 140)	131 (119, 146)
Diastolic blood pressure (mmhg)	74 (69, 82)	74 (67, 81)	74 (69, 82)	75 (70, 83)	76 (69, 82)
Heart rate (beats/minute)	76 (67, 86)	75 (65, 83)	75 (66, 88)	75 (69, 85)	80 (70, 91)
Clinical Covariates					
Coronary artery disease	102 (20%)	34 (27%)	30 (25%)	24 (17%)	14 (12%)
Congestive heart failure	212 (42%)	67 (52%)	63 (52%)	50 (36%)	32 (27%)
Stroke/TIA	90 (18%)	25 (20%)	17 (14%)	23 (17%)	25 (21%)
Diabetes mellitus	221 (44%)	53 (41%)	56 (46%)	55 (40%)	57 (49%)
Hypertension	392 (76%)	103 (78%)	101 (83%)	98 (70%)	90 (74%)

Chronic kidney disease	139 (28%)	35 (27%)	33 (27%)	40 (29%)	31 (26%)
COPD	126 (25%)	41 (32%)	36 (30%)	28 (20%)	21 (18%)
CHA2DS2VASc score	3 (2, 5)	3 (2, 5)	3 (2, 5)	3 (2, 4)	3 (2, 4)
Type of AF					
Paroxysmal	411 (80%)	104 (79%)	92 (76%)	112 (79%)	103 (84%)
Persistent	105 (20%)	28 (21%)	29 (24%)	29 (21%)	19 (16%)
Previous Cardiac Procedures					
Pacemaker or defibrillator implant	24 (4.7%)	7 (5.3%)	5 (4.1%)	7 (5.0%)	5 (4.1%)
Electrical direct-current cardioversion	60 (12%)	18 (14%)	11 (9.1%)	23 (16%)	8 (6.6%)
Catheter ablation	48 (9.3%)	13 (9.8%)	12 (9.9%)	11 (7.8%)	12 (9.8%)
Baseline Treatment Strategy					
Anticoagulant agents	383 (74%)	103 (78%)	93 (77%)	105 (74%)	82 (67%)
Rate-control medications	410 (79%)	104 (79%)	104 (86%)	109 (77%)	93 (76%)
Rhythm-control medications	161 (31%)	48 (36%)	37 (31%)	39 (28%)	37 (30%)
Rhythm-control strategy	188 (36%)	56 (42%)	45 (37%)	44 (31%)	43 (35%)

Abbreviations: AF = atrial fibrillation, AFEQT = atrial fibrillation effect on quality-of-life, BMI = body mass index, calculated as weight in kilograms divided by height in meters squared, CABG = coronary artery bypass grafting, CI = confidence interval, CKD = chronic kidney disease, COPD = chronic obstructive pulmonary disease, CVA = cerebrovascular event or stroke, HTN = hypertension, NDI = neighborhood deprivation index, PCI = percutaneous coronary intervention, SDoH = social determinants of health, TIA = transient ischemic attack

[a] Median (Q1, Q3); n (%)

Table S2: ***Baseline Cohort Description by AFEQT Score***

	**Overall**<br>N = 875[a]	**Q1 (5 - 58)**<br>N = 219[a]	**Q2 (58 - 78)**<br>N = 216[a]	**Q3 (78 - 93)**<br>N = 220[a]	**Q4 (93 - 100)**<br>N = 220[a]
Sociodemographics					
Age (years)	64 (57, 72)	62 (57, 69)	63 (55, 71)	64 (59, 73)	67 (59, 75)
Sex					
Female	314 (39%)	88 (44%)	82 (41%)	65 (32%)	79 (39%)
Male	493 (61%)	111 (56%)	116 (59%)	140 (68%)	126 (61%)
Race & Ethnicity					
Non-Hispanic White	353 (40%)	86 (39%)	84 (39%)	94 (43%)	89 (40%)
Hispanic/Latinx	161 (18%)	39 (18%)	37 (17%)	50 (23%)	35 (16%)
Non-Hispanic Black	361 (41%)	94 (43%)	95 (44%)	76 (35%)	96 (44%)
Neighborhood deprivation index by quartile					
Least deprivation	111 (13%)	23 (11%)	27 (13%)	30 (14%)	31 (14%)
Below average deprivation	102 (12%)	28 (13%)	19 (8.8%)	26 (12%)	29 (13%)
Above average deprivation	172 (20%)	54 (25%)	38 (18%)	41 (19%)	39 (18%)
Most deprivation	490 (56%)	114 (52%)	132 (61%)	123 (56%)	121 (55%)
Insurance Class					
Private	92 (11%)	16 (7.3%)	26 (12%)	25 (11%)	25 (11%)
Public	599 (68%)	152 (69%)	149 (69%)	144 (65%)	154 (70%)
Self-Pay	184 (21%)	51 (23%)	41 (19%)	51 (23%)	41 (19%)
Language Group					
English	705 (81%)	177 (81%)	175 (81%)	175 (80%)	178 (81%)
Other primary language	82 (9.4%)	22 (10%)	21 (9.7%)	20 (9.1%)	19 (8.6%)
Spanish	88 (10%)	20 (9.1%)	20 (9.3%)	25 (11%)	23 (10%)
Marital Status					
Married or cohabitating	346 (40%)	82 (37%)	88 (41%)	93 (42%)	83 (38%)
Divorced, separated, or widowed	186 (21%)	46 (21%)	45 (21%)	48 (22%)	47 (21%)
Single or never-married	343 (39%)	91 (42%)	83 (38%)	79 (36%)	90 (41%)
Vital Signs					
Body mass index (kg/m^2)	30 (26, 36)	30 (26, 36)	31 (26, 37)	31 (28, 35)	30 (27, 34)
Systolic blood pressure (mmhg)	131 (119, 142)	126 (116, 138)	131 (120, 143)	133 (124, 140)	132 (120, 146)
Diastolic blood pressure (mmhg)	75 (69, 82)	73 (67, 81)	76 (70, 83)	76 (70, 83)	76 (72, 83)
Heart rate (beats/minute)	76 (68, 85)	76 (67, 85)	76 (70, 85)	76 (68, 84)	79 (70, 88)
Clinical Covariates					

Coronary artery disease	188 (23%)	57 (28%)	55 (27%)	39 (19%)	37 (18%)
Congestive heart failure	345 (42%)	112 (54%)	100 (49%)	72 (35%)	61 (30%)
Stroke/TIA	144 (18%)	36 (17%)	31 (15%)	39 (19%)	38 (19%)
Diabetes mellitus	352 (43%)	89 (43%)	89 (43%)	84 (40%)	90 (45%)
Hypertension	610 (70%)	156 (71%)	161 (75%)	140 (64%)	153 (70%)
Chronic kidney disease	220 (27%)	58 (28%)	54 (26%)	55 (26%)	53 (26%)
COPD	223 (27%)	62 (30%)	73 (36%)	45 (22%)	43 (21%)
CHA2DS2VASc score	3 (1, 4)	3 (1, 4)	3 (1, 4)	3 (1, 4)	3 (1, 4)
Type of AF					
Paroxysmal	696 (80%)	172 (79%)	166 (77%)	178 (81%)	180 (82%)
Persistent	179 (20%)	47 (21%)	50 (23%)	42 (19%)	40 (18%)
Previous Cardiac Procedures					
Pacemaker or defibrillator implant	42 (4.8%)	13 (5.9%)	9 (4.2%)	10 (4.5%)	10 (4.5%)
Electrical direct-current cardioversion	91 (10%)	26 (12%)	22 (10%)	29 (13%)	14 (6.4%)
Catheter ablation	64 (7.3%)	16 (7.3%)	16 (7.4%)	15 (6.8%)	17 (7.7%)
Baseline Treatment Strategy					
Anticoagulant agents	550 (63%)	146 (67%)	137 (63%)	139 (63%)	128 (58%)
Rate-control medications	627 (72%)	160 (73%)	163 (75%)	154 (70%)	150 (68%)
Rhythm-control medications	217 (25%)	59 (27%)	51 (24%)	54 (25%)	53 (24%)
Rhythm-control strategy	256 (29%)	69 (32%)	62 (29%)	61 (28%)	64 (29%)

Abbreviations: AF = atrial fibrillation, AFEQT = atrial fibrillation effect on quality-of-life, BMI = body mass index, calculated as weight in kilograms divided by height in meters squared, CABG = coronary artery bypass grafting, CI = confidence interval, CKD = chronic kidney disease, COPD = chronic obstructive pulmonary disease, CVA = cerebrovascular event or stroke, HTN = hypertension, NDI = neighborhood deprivation index, PCI = percutaneous coronary intervention, SDoH = social determinants of health, TIA = transient ischemic attack

[a] Median (Q1, Q3); n (%)

Table S3: ***Missing Cohort by AFEQT Score***

	**Overall** N = 359[a]	**Q1 (5 - 58)** N = 87[a]	**Q2 (58 - 78)** N = 95[a]	**Q3 (78 - 93)** N = 79[a]	**Q4 (93 - 100)** N = 98[a]
Sociodemographics					
Age (years)	64 (55, 72)	64 (57, 70)	61 (53, 71)	64 (54, 72)	67 (59, 75)
Sex					
Female	129 (44%)	32 (48%)	39 (51%)	21 (33%)	37 (45%)
Male	162 (56%)	35 (52%)	38 (49%)	43 (67%)	46 (55%)
Race & Ethnicity					
Non-Hispanic White	169 (47%)	41 (47%)	43 (45%)	42 (53%)	43 (44%)
Hispanic/Latinx	36 (10%)	10 (11%)	7 (7.4%)	11 (14%)	8 (8.2%)
Non-Hispanic Black	154 (43%)	36 (41%)	45 (47%)	26 (33%)	47 (48%)
Neighborhood deprivation index by quartile					
Least deprivation	42 (12%)	9 (10%)	10 (11%)	11 (14%)	12 (12%)
Below average deprivation	39 (11%)	7 (8.0%)	6 (6.3%)	12 (15%)	14 (14%)
Above average deprivation	76 (21%)	24 (28%)	19 (20%)	14 (18%)	19 (19%)
Most deprivation	202 (56%)	47 (54%)	60 (63%)	42 (53%)	53 (54%)
Insurance Class					
Private	44 (12%)	5 (5.7%)	12 (13%)	16 (20%)	11 (11%)
Public	246 (69%)	61 (70%)	67 (71%)	46 (58%)	72 (73%)
Self-Pay	69 (19%)	21 (24%)	16 (17%)	17 (22%)	15 (15%)
Language Group					
English	260 (72%)	60 (69%)	72 (76%)	54 (68%)	74 (76%)
Other primary language	78 (22%)	22 (25%)	19 (20%)	19 (24%)	18 (18%)
Spanish	21 (5.8%)	5 (5.7%)	4 (4.2%)	6 (7.6%)	6 (6.1%)
Marital Status					
Married or cohabitating	130 (36%)	29 (33%)	40 (42%)	25 (32%)	36 (37%)
Divorced, separated, or widowed	77 (21%)	22 (25%)	18 (19%)	16 (20%)	21 (21%)
Single or never-married	152 (42%)	36 (41%)	37 (39%)	38 (48%)	41 (42%)
Vital Signs					
Body mass index (kg/m^2)	32 (27, 37)	33 (26, 39)	31 (26, 36)	31 (28, 38)	31 (27, 34)
Systolic blood pressure (mmhg)	130 (119, 145)	125 (113, 142)	131 (120, 146)	131 (123, 138)	132 (121, 145)
Diastolic blood pressure (mmhg)	76 (70, 82)	72 (67, 80)	79 (71, 83)	77 (69, 82)	77 (73, 84)
Heart rate (beats/minute)	77 (70, 85)	77 (72, 87)	76 (71, 83)	77 (68, 84)	77 (68, 87)
Clinical Covariates					
Coronary artery disease	86 (27%)	23 (29%)	25 (30%)	15 (22%)	23 (27%)
Congestive heart failure	133 (42%)	45 (57%)	37 (44%)	22 (32%)	29 (34%)
Stroke/TIA	54 (17%)	11 (14%)	14 (17%)	16 (23%)	13 (15%)
Diabetes mellitus	131 (41%)	36 (46%)	33 (39%)	29 (42%)	33 (39%)
Hypertension	218 (61%)	53 (61%)	60 (63%)	42 (53%)	63 (64%)

Chronic kidney disease	81 (26%)	23 (29%)	21 (25%)	15 (22%)	22 (26%)
COPD	97 (31%)	21 (27%)	37 (44%)	17 (25%)	22 (26%)
CHA2DS2VASc score	2 (1, 4)	2 (1, 4)	2 (1, 4)	2 (1, 4)	2 (1, 4)
Type of AF					
Paroxysmal	285 (79%)	68 (78%)	74 (78%)	66 (84%)	77 (79%)
Persistent	74 (21%)	19 (22%)	21 (22%)	13 (16%)	21 (21%)
Previous Cardiac Procedures					
Pacemaker or defibrillator implant	18 (5.0%)	6 (6.9%)	4 (4.2%)	3 (3.8%)	5 (5.1%)
Electrical direct-current cardioversion	31 (8.6%)	8 (9.2%)	11 (12%)	6 (7.6%)	6 (6.1%)
Catheter ablation	16 (4.5%)	3 (3.4%)	4 (4.2%)	4 (5.1%)	5 (5.1%)
Baseline Treatment Strategy					
Anticoagulant agents	167 (47%)	43 (49%)	44 (46%)	34 (43%)	46 (47%)
Rate-control medications	217 (60%)	56 (64%)	59 (62%)	45 (57%)	57 (58%)
Rhythm-control medications	56 (16%)	11 (13%)	14 (15%)	15 (19%)	16 (16%)
Rhythm-control strategy	68 (19%)	13 (15%)	17 (18%)	17 (22%)	21 (21%)

Abbreviations: AF = atrial fibrillation, AFEQT = atrial fibrillation effect on quality-of-life, BMI = body mass index, calculated as weight in kilograms divided by height in meters squared, CABG = coronary artery bypass grafting, CI = confidence interval, CKD = chronic kidney disease, COPD = chronic obstructive pulmonary disease, CVA = cerebrovascular event or stroke, HTN = hypertension, NDI = neighborhood deprivation index, PCI = percutaneous coronary intervention, SDoH = social determinants of health, TIA = transient ischemic attack

[a] Median (Q1, Q3); n (%)

Table S4: ***Longitudinal Cohort Description by Race & Ethnicity***

	Overall (n = 516)[a]	Non-Hispanic White (n = 184)[a]	Hispanic/Latinx (n = 125)[a]	Non-Hispanic Black (n = 207)[a]
Sociodemographics				
Age (years)	64 (58, 71)	65 (60, 71)	65 (57, 73)	63 (55, 71)
Sex				
Female	185 (36%)	38 (21%)	53 (42%)	94 (45%)
Male	331 (64%)	146 (79%)	72 (58%)	113 (55%)
Neighborhood deprivation index by quartile				
Least deprivation	69 (13%)	52 (28%)	7 (5.6%)	10 (4.8%)
Below average deprivation	63 (12%)	35 (19%)	14 (11%)	14 (6.8%)
Above average deprivation	96 (19%)	44 (24%)	15 (12%)	37 (18%)
Most deprivation	288 (56%)	53 (29%)	89 (71%)	146 (71%)
Insurance Class				
Private	48 (9.3%)	17 (9.2%)	10 (8.0%)	21 (10%)
Public	353 (68%)	134 (73%)	77 (62%)	142 (69%)
Self-Pay	115 (22%)	33 (18%)	38 (30%)	44 (21%)
Language Group				
English	445 (86%)	178 (97%)	60 (48%)	207 (100%)
Other primary language	4 (0.8%)	3 (1.6%)	1 (0.8%)	0 (0%)
Spanish	67 (13%)	3 (1.6%)	64 (51%)	0 (0%)
Marital Status				
Married or cohabitating	216 (42%)	85 (46%)	57 (46%)	74 (36%)
Divorced, separated, or widowed	109 (21%)	37 (20%)	29 (23%)	43 (21%)
Single or never-married	191 (37%)	62 (34%)	39 (31%)	90 (43%)
Clinical Covariates				
Coronary artery disease	102 (20%)	39 (22%)	23 (19%)	40 (20%)
Congestive heart failure	212 (42%)	60 (33%)	53 (44%)	99 (48%)
Stroke/TIA	90 (18%)	29 (16%)	19 (16%)	42 (20%)
Diabetes mellitus	221 (44%)	68 (38%)	61 (51%)	92 (45%)
Hypertension	392 (76%)	139 (76%)	92 (74%)	161 (78%)
Chronic kidney disease	139 (28%)	36 (20%)	40 (33%)	63 (31%)
COPD	126 (25%)	36 (20%)	31 (26%)	59 (29%)
CHA2DS2VASc score	3 (2, 5)	3 (2, 4)	3 (2, 5)	3 (2, 5)
Type of AF				
Paroxysmal	411 (80%)	142 (77%)	102 (82%)	167 (81%)
Persistent	105 (20%)	42 (23%)	23 (18%)	40 (19%)
Previous Cardiac Procedures				
Pacemaker or defibrillator implant	24 (4.7%)	9 (4.9%)	5 (4.0%)	10 (4.8%)
Electrical direct-current cardioversion	60 (12%)	27 (15%)	15 (12%)	18 (8.7%)
Catheter ablation	48 (9.3%)	17 (9.2%)	11 (8.8%)	20 (9.7%)
Baseline Treatment Strategy				
Anticoagulant agents	383 (74%)	132 (72%)	87 (70%)	164 (79%)
Rate-control medications	410 (79%)	138 (75%)	104 (83%)	168 (81%)
Rhythm-control medications	161 (31%)	65 (35%)	41 (33%)	55 (27%)
Rhythm-control strategy	188 (36%)	73 (40%)	46 (37%)	69 (33%)

Abbreviations: AF = atrial fibrillation, AFEQT = atrial fibrillation effect on quality-of-life, BMI = body mass index, calculated as weight in kilograms divided by height in meters squared, CABG = coronary artery bypass grafting, CI = confidence interval, CKD = chronic kidney disease, COPD = chronic obstructive pulmonary disease, CVA = cerebrovascular event or stroke, HTN = hypertension, NDI = neighborhood deprivation index, PCI = percutaneous coronary intervention, SDoH = social determinants of health, TIA = transient ischemic attack

[a] Median (Q1, Q3); n (%)

Table S5: ***Longitudinal Cohort Treatment Characteristics by Race and Ethnicity***

	Overall (n = 516)[a]	Non-Hispanic White (n = 184)[a]	Hispanic/Latinx (n = 125)[a]	Non-Hispanic Black (n = 207)[a]
Follow-up Medications				
Antiplatelet agents	212 (41%)	59 (32%)	61 (49%)	92 (44%)
Anticoagulant agents	353 (68%)	107 (58%)	96 (77%)	150 (72%)
Sodium channel blockers	71 (16%)	28 (20%)	20 (16%)	23 (13%)
Beta blockers	346 (67%)	109 (59%)	98 (78%)	139 (67%)
Potassium channel blockers	134 (26%)	47 (26%)	39 (31%)	48 (23%)
Calcium channel blockers	56 (11%)	16 (8.7%)	15 (12%)	25 (12%)
Follow-up Treatment Strategy				
Rate-control medications	360 (70%)	113 (61%)	101 (81%)	146 (71%)
Rhythm-control medications	179 (40%)	65 (45%)	51 (41%)	63 (36%)
Rhythm-control strategy	260 (55%)	102 (61%)	64 (52%)	94 (50%)
Catheter ablation	68 (13%)	29 (16%)	16 (13%)	23 (11%)
Electrical direct-current cardioversion	54 (10%)	22 (12%)	12 (9.6%)	20 (9.7%)
Baseline AFEQT Scores				
Overall	79 (57, 92)	81 (58, 93)	81 (62, 91)	76 (54, 93)
Symptoms	88 (67, 100)	92 (67, 100)	88 (67, 100)	88 (63, 100)
Daily activities	73 (44, 96)	77 (46, 96)	76 (50, 96)	71 (42, 96)
Treatment concerns	83 (61, 100)	86 (64, 100)	89 (67, 100)	83 (53, 100)
Changes in AFEQT Scores				
Overall (change)	5 (-5, 20)	7 (-2, 18)	4 (-5, 21)	4 (-8, 21)
Symptoms (change)	0 (-4, 17)	0 (0, 21)	0 (-2, 17)	0 (-8, 18)
Daily activities (change)	2 (-8, 25)	2 (-8, 22)	5 (-4, 27)	2 (-13, 27)
Treatment concerns (change)	0 (-6, 22)	3 (0, 22)	0 (-10, 18)	0 (-7, 25)
Major Adverse Cardiovascular Events				
Acute coronary syndrome	56 (13%)	13 (9.0%)	13 (11%)	30 (17%)
Coronary revascularization	18 (3.5%)	7 (3.8%)	1 (0.8%)	10 (4.8%)
Acute decompensated heart failure	61 (14%)	16 (11%)	19 (16%)	26 (14%)
Acute ischemic stroke	53 (13%)	12 (9.1%)	16 (14%)	25 (14%)
All-cause mortality	47 (9.1%)	13 (7.1%)	15 (12%)	19 (9.2%)

Abbreviations: AF = atrial fibrillation, AFEQT = atrial fibrillation effect on quality-of-life, BMI = body mass index, calculated as weight in kilograms divided by height in meters squared, CABG = coronary artery bypass grafting, CI = confidence interval, CKD = chronic kidney disease, COPD = chronic obstructive pulmonary disease, CVA = cerebrovascular event or stroke, HTN = hypertension, NDI = neighborhood deprivation index, PCI = percutaneous coronary intervention, SDoH = social determinants of health, TIA = transient ischemic attack

[a] Median (Q1, Q3); n (%)

Table S6: ***AFEQT Treatment Domain and Social Determinants of Health with Traditional Risk Factors***

	NDI Quartile (Least deprivation)[a]			Race & Ethnicity (Non-Hispanic White)[b]		Sex (Female)	Insurance (Private)[c]		Language (English)[d]		Marital Status (Married or cohabitating)	
	Below average deprivation	Above average deprivation	Most deprivation	Hispanic/Latinx	Non-Hispanic Black	Male	Public	Self-Pay	Other primary language	Spanish	Divorced, separated, or widowed	Single or never-married
Change in Treatment AFEQT Score[e]												
Model 1 = Adjustment for baseline AFEQT[f]	−2.6 (−9.4, 4.3)	−2.6 (−8.7, 3.6)	−4.5 (−9.8, 0.7)	−5.1 (−9.6, −0.6)	−7.1 (−11.0, −3.1)	2.9 (−0.7, 6.5)	3.4 (−2.5, 9.4)	3.7 (−3.0, 10.4)	−5.1 (−24.7, 14.5)	−3.3 (−8.4, 1.8)	0.9 (−3.7, 5.4)	1.1 (−2.8, 5.0)
Model 2 = Model 1 + age + BMI + sociodemographic factors[g]	−2.3 (−9.2, 4.6)	−2.2 (−8.4, 4.0)	−4.3 (−9.5, 1.0)	−5.1 (−9.5, −0.6)	−6.9 (−10.8, −3.0)	3.1 (−0.5, 6.7)	2.9 (−3.2, 9.0)	3.0 (−3.8, 9.8)	−4.6 (−24.2, 15.0)	−3.8 (−9.0, 1.3)	0.2 (−4.5, 4.9)	1.7 (−2.3, 5.7)
Model 3 = Model 2 + cardiovascular risk factors[h]	−2.6 (−9.6, 4.5)	−2.2 (−8.5, 4.1)	−4.4 (−9.8, 1.0)	−5.9 (−10.5, −1.3)	−7.3 (−11.3, −3.3)	3.4 (−0.3, 7.1)	2.8 (−3.3, 9.0)	2.5 (−4.5, 9.4)	−3.8 (−23.6, 16.0)	−4.4 (−9.8, 0.9)	0.1 (−4.7, 4.9)	2.1 (−2.0, 6.1)
Model 4 = Model 3 + major cardiovascular adverse events[i]	−3.0 (−10.0, 4.0)	−2.7 (−9.0, 3.6)	−4.6 (−10.0, 0.8)	−5.7 (−10.3, −1.1)	−7.1 (−11.1, −3.1)	2.8 (−0.9, 6.5)	3.2 (−2.9, 9.4)	3.2 (−3.7, 10.2)	−3.2 (−23.0, 16.6)	−4.1 (−9.5, 1.2)	0.6 (−4.3, 5.4)	2.9 (−1.2, 7.0)
Model 5 = Model 4 + treatment strategy	−3.4 (−10.4, 3.6)	−2.8 (−9.1, 3.5)	−5.1 (−10.5, 0.3)	−5.8 (−10.4, −1.2)	−7.5 (−11.5, −3.4)	2.8 (−0.9, 6.5)	3.1 (−3.0, 9.3)	3.1 (−3.8, 10.1)	−2.6 (−22.4, 17.2)	−4.1 (−9.4, 1.3)	0.3 (−4.5, 5.1)	2.7 (−1.4, 6.8)

Abbreviations: AF = atrial fibrillation, AFEQT = atrial fibrillation effect on quality-of-life, BMI = body mass index, calculated as weight in kilograms divided by height in meters squared, CABG = coronary artery bypass grafting, CI = confidence interval, CKD = chronic kidney disease, COPD = chronic obstructive pulmonary disease, CVA = cerebrovascular event or stroke, HTN = hypertension, NDI = neighborhood deprivation index, PCI = percutaneous coronary intervention, SDoH = social determinants of health, TIA = transient ischemic attack

[a] The NDI quartiles were determined based on comparison of the NDI at the selected census track compared to the national average. The referent group was the bottom quartile of NDI, described as tracks with the least deprivation (n = 69). The below average (n = 63), above average (n = 96), and most deprivation (n = 288) quartiles were considered as ordinal categories for comparison.

[b] These categories were determined by self-report. Non-Hispanic Black (n = 207) and Hispanic/Latinx (n = 125) were compared with Non-Hispanic White (n = 184) participants as the reference for all models.

[c] Public insurance (n = 353) and self-pay (n = 115) insurance were compared against private (n = 48) insurance, which was the reference group for all models.

[d] Language was confirmed via patient interview. Spanish (n = 67) was selected if a translator was required for the interview, or was categorized as 'Other primary langauge' (n = 4). The referent language for models was English (n= 445).

[e] Linear mixed effects models for the continuous change in AFEQT score, with random effects for site and subtype of AF. Beta estimates (95% CI) are presented.

[f] As the outcome was defined as change in AFEQT over study period, all models adjusted for baseline AFEQT scores.

[g] Sociodemographic factors included age, race & ethnicity, and sex, when not included as the primary exposure).

[h] Cardiovascular risk factors included obesity, smoking, hypertension, diabetes, kidney disease, and hyperlipidemia.

[i] The prevalent major cardiovascular adverse events included coronary artery disease, cerebrovascular accident (stroke or TIA), congestive heart failure, or peripheral vascular disease.

Table S7: ***AFEQT Activity Domain and Social Determinants of Health with Traditional Risk Factors***

	NDI Quartile (Least deprivation)[a]			Race & Ethnicity (Non-Hispanic White)[b]		Sex (Female)	Insurance (Private)[c]		Language (English)[d]		Marital Status (Married or cohabitating)	
	Below average deprivation	Above average deprivation	Most deprivation	Hispanic/Latinx	Non-Hispanic Black	Male	Public	Self-Pay	Other primary language	Spanish	Divorced, separated, or widowed	Single or never-married
Change in Activities AFEQT Score[e]												
Model 1 = Adjustment for baseline AFEQT[f]	−2.7 (−10.9, 5.6)	−6.4 (−13.9, 1.0)	−7.4 (−13.7, −1.0)	2.5 (−3.0, 8.0)	−3.4 (−8.2, 1.3)	0.8 (−3.5, 5.2)	−4.2 (−11.5, 3.1)	−5.8 (−14.0, 2.3)	11.8 (−11.8, 35.4)	8.3 (2.2, 14.5)	−3.1 (−8.6, 2.5)	−0.8 (−5.6, 3.9)
Model 2 = Model 1 + age + BMI + sociodemographic factors[g]	−2.4 (−10.7, 5.9)	−6.0 (−13.5, 1.5)	−7.1 (−13.5, −0.8)	2.5 (−2.9, 8.0)	−3.2 (−8.0, 1.6)	1.0 (−3.4, 5.3)	−5.1 (−12.5, 2.3)	−6.9 (−15.2, 1.3)	12.0 (−11.7, 35.6)	7.9 (1.7, 14.2)	−3.9 (−9.6, 1.8)	−0.1 (−4.9, 4.7)
Model 3 = Model 2 + cardiovascular risk factors[h]	−2.0 (−10.5, 6.5)	−5.4 (−13.1, 2.2)	−6.9 (−13.4, −0.4)	2.3 (−3.3, 8.0)	−3.5 (−8.4, 1.4)	0.9 (−3.6, 5.3)	−5.2 (−12.6, 2.3)	−6.4 (−14.8, 2.0)	11.5 (−12.3, 35.3)	8.7 (2.3, 15.2)	−3.5 (−9.4, 2.3)	0.2 (−4.7, 5.1)
Model 4 = Model 3 + major cardiovascular adverse events[i]	−2.3 (−10.8, 6.1)	−5.6 (−13.2, 2.0)	−6.7 (−13.2, −0.2)	2.9 (−2.8, 8.5)	−2.9 (−7.8, 2.1)	0.2 (−4.2, 4.7)	−4.6 (−12.0, 2.9)	−5.6 (−14.1, 2.8)	11.2 (−12.7, 35.0)	9.0 (2.6, 15.5)	−2.7 (−8.6, 3.2)	1.1 (−3.8, 6.1)
Model 5 = Model 4 + treatment strategy	−2.2 (−10.6, 6.3)	−5.6 (−13.2, 2.0)	−6.5 (−13.0, 0.0)	2.9 (−2.7, 8.6)	−2.7 (−7.6, 2.3)	0.2 (−4.2, 4.7)	−4.5 (−12.0, 2.9)	−5.5 (−14.0, 2.9)	10.8 (−13.0, 34.7)	9.0 (2.5, 15.5)	−2.5 (−8.4, 3.4)	1.2 (−3.7, 6.2)

Abbreviations: AF = atrial fibrillation, AFEQT = atrial fibrillation effect on quality-of-life, BMI = body mass index, calculated as weight in kilograms divided by height in meters squared, CABG = coronary artery bypass grafting, CI = confidence interval, CKD = chronic kidney disease, COPD = chronic obstructive pulmonary disease, CVA = cerebrovascular event or stroke, HTN = hypertension, NDI = neighborhood deprivation index, PCI = percutaneous coronary intervention, SDoH = social determinants of health, TIA = transient ischemic attack

[a] The NDI quartiles were determined based on comparison of the NDI at the selected census track compared to the national average. The referent group was the bottom quartile of NDI, described as tracks with the least deprivation (n = 69). The below average (n = 63), above average (n = 96), and most deprivation (n = 288) quartiles were considered as ordinal categories for comparison.

[b] These categories were determined by self-report. Non-Hispanic Black (n = 207) and Hispanic/Latinx (n = 125) were compared with Non-Hispanic White (n = 184) participants as the reference for all models.

[c] Public insurance (n = 353) and self-pay (n = 115) insurance were compared against private (n = 48) insurance, which was the reference group for all models.

[d] Language was confirmed via patient interview. Spanish (n = 67) was selected if a translator was required for the interview, or was categorized as 'Other primary langauge' (n = 4). The referent language for models was English (n= 445).

[e] Linear mixed effects models for the continuous change in AFEQT score, with random effects for site and subtype of AF. Beta estimates (95% CI) are presented.

[f] As the outcome was defined as change in AFEQT over study period, all models adjusted for baseline AFEQT scores.

[g] Sociodemographic factors included age, race & ethnicity, and sex, when not included as the primary exposure).

[h] Cardiovascular risk factors included obesity, smoking, hypertension, diabetes, kidney disease, and hyperlipidemia.

[i] The prevalent major cardiovascular adverse events included coronary artery disease, cerebrovascular accident (stroke or TIA), congestive heart failure, or peripheral vascular disease.

Table S8: ***AFEQT Symptoms Domain and Social Determinants of Health with Traditional Risk Factors***

	NDI Quartile (Least deprivation)[a]			Race & Ethnicity (Non-Hispanic White)[b]		Sex (Female)	Insurance (Private)[c]		Language (English)[d]		Marital Status (Married or cohabitating)	
	Below average deprivation	Above average deprivation	Most deprivation	Hispanic/Latinx	Non-Hispanic Black	Male	Public	Self-Pay	Other primary language	Spanish	Divorced, separated, or widowed	Single or never-married
Change in Symptoms AFEQT Score[e]												
Model 1 = Adjustment for baseline AFEQT[f]	−2.8 (−9.8, 4.3)	−4.0 (−10.4, 2.4)	−5.6 (−11.0, −0.2)	−2.1 (−6.8, 2.5)	−3.6 (−7.7, 0.5)	1.5 (−2.2, 5.2)	1.5 (−4.7, 7.7)	1.9 (−5.1, 8.8)	2.1 (−17.8, 22.1)	3.4 (−1.9, 8.6)	−4.6 (−9.3, 0.1)	−0.3 (−4.3, 3.7)
Model 2 = Model 1 + age + BMI + sociodemographic factors[g]	−2.2 (−9.3, 4.8)	−3.2 (−9.7, 3.2)	−5.1 (−10.5, 0.3)	−2.1 (−6.7, 2.6)	−3.2 (−7.3, 0.9)	1.8 (−1.9, 5.5)	0.2 (−6.0, 6.5)	0.3 (−6.7, 7.3)	2.6 (−17.3, 22.5)	2.4 (−2.8, 7.7)	−6.2 (−11.0, −1.5)	1.1 (−2.9, 5.1)
Model 3 = Model 2 + cardiovascular risk factors[h]	−2.5 (−9.6, 4.7)	−3.6 (−10.2, 2.9)	−5.4 (−11.0, 0.1)	−2.3 (−7.1, 2.4)	−3.2 (−7.4, 0.9)	1.8 (−1.9, 5.6)	−0.1 (−6.4, 6.3)	0.0 (−7.2, 7.2)	3.6 (−16.6, 23.8)	2.1 (−3.4, 7.6)	−6.5 (−11.4, −1.6)	1.1 (−3.0, 5.2)
Model 4 = Model 3 + major cardiovascular adverse events[i]	−2.9 (−10.1, 4.2)	−4.1 (−10.6, 2.5)	−5.5 (−11.1, 0.0)	−1.9 (−6.6, 2.9)	−2.8 (−7.0, 1.4)	1.1 (−2.7, 4.9)	0.5 (−5.9, 6.8)	0.8 (−6.4, 8.0)	4.3 (−15.8, 24.5)	2.5 (−3.0, 7.9)	−6.0 (−10.9, −1.0)	1.9 (−2.2, 6.1)
Model 5 = Model 4 + treatment strategy	−2.8 (−10.0, 4.3)	−4.1 (−10.6, 2.5)	−5.4 (−10.9, 0.2)	−1.8 (−6.6, 2.9)	−2.7 (−6.9, 1.5)	1.1 (−2.7, 4.9)	0.5 (−5.8, 6.9)	0.9 (−6.3, 8.1)	4.1 (−16.1, 24.3)	2.4 (−3.1, 7.9)	−5.8 (−10.8, −0.9)	2.0 (−2.2, 6.1)

Abbreviations: AF = atrial fibrillation, AFEQT = atrial fibrillation effect on quality-of-life, BMI = body mass index, calculated as weight in kilograms divided by height in meters squared, CABG = coronary artery bypass grafting, CI = confidence interval, CKD = chronic kidney disease, COPD = chronic obstructive pulmonary disease, CVA = cerebrovascular event or stroke, HTN = hypertension, NDI = neighborhood deprivation index, PCI = percutaneous coronary intervention, SDoH = social determinants of health, TIA = transient ischemic attack

[a] The NDI quartiles were determined based on comparison of the NDI at the selected census track compared to the national average. The referent group was the bottom quartile of NDI, described as tracks with the least deprivation (n = 69). The below average (n = 63), above average (n = 96), and most deprivation (n = 288) quartiles were considered as ordinal categories for comparison.

[b] These categories were determined by self-report. Non-Hispanic Black (n = 207) and Hispanic/Latinx (n = 125) were compared with Non-Hispanic White (n = 184) participants as the reference for all models.

[c] Public insurance (n = 353) and self-pay (n = 115) insurance were compared against private (n = 48) insurance, which was the reference group for all models.

[d] Language was confirmed via patient interview. Spanish (n = 67) was selected if a translator was required for the interview, or was categorized as 'Other primary langauge' (n = 4). The referent language for models was English (n= 445).

[e] Linear mixed effects models for the continuous change in AFEQT score, with random effects for site and subtype of AF. Beta estimates (95% CI) are presented.

[f] As the outcome was defined as change in AFEQT over study period, all models adjusted for baseline AFEQT scores.

[g] Sociodemographic factors included age, race & ethnicity, and sex, when not included as the primary exposure).

[h] Cardiovascular risk factors included obesity, smoking, hypertension, diabetes, kidney disease, and hyperlipidemia.

[i] The prevalent major cardiovascular adverse events included coronary artery disease, cerebrovascular accident (stroke or TIA), congestive heart failure, or peripheral vascular disease.

Table S9: ***AFEQT Scores and Social Determinants at Baseline***

	NDI Quartile (Least deprivation)[a]			Race & Ethnicity (Non-Hispanic White)[b]		Sex (Female)	Insurance (Private)[c]		Language (English)[d]		Marital Status (Married or cohabitating)	
	Below average deprivation	Above average deprivation	Most deprivation	Hispanic/Latinx	Non-Hispanic Black	Male	Public	Self-Pay	Other primary language	Spanish	Divorced, separated, or widowed	Single or never-married
Baseline Total AFEQT Score												
Model 1 = Social determinant	−5.2 (−13.1, 2.7)	−4.0 (−11.2, 3.2)	−1.7 (−7.8, 4.4)	0.0 (−5.3, 5.2)	−3.4 (−8.0, 1.2)	4.1 (−0.1, 8.2)	−0.4 (−7.4, 6.6)	−0.5 (−8.3, 7.3)	10.0 (−12.7, 32.8)	3.1 (−2.9, 9.0)	0.9 (−4.4, 6.2)	−2.7 (−7.2, 1.8)
Model 2 = Model 1 + age + BMI[e]	−4.2 (−12.1, 3.6)	−2.6 (−9.7, 4.6)	−0.7 (−6.7, 5.4)	0.2 (−5.0, 5.4)	−2.6 (−7.2, 1.9)	4.4 (0.3, 8.5)	−2.5 (−9.5, 4.5)	−3.0 (−10.9, 4.8)	11.0 (−11.5, 33.6)	1.6 (−4.4, 7.6)	−1.0 (−6.4, 4.4)	−1.2 (−5.8, 3.4)
Model 3 = Model 2 + cardiovascular risk factors[f]	−5.5 (−13.4, 2.4)	−1.2 (−8.4, 5.9)	0.0 (−6.1, 6.1)	0.3 (−5.0, 5.6)	−2.4 (−7.0, 2.2)	3.8 (−0.3, 8.0)	−2.5 (−9.5, 4.5)	−2.9 (−10.8, 4.9)	14.5 (−7.9, 37.0)	1.5 (−4.6, 7.5)	0.1 (−5.4, 5.6)	−1.4 (−5.9, 3.2)
Model 4 = Model 3 + major cardiovascular adverse events[g]	−5.5 (−13.3, 2.2)	−0.4 (−7.5, 6.6)	0.4 (−5.6, 6.4)	0.4 (−4.8, 5.6)	−2.2 (−6.8, 2.4)	4.6 (0.4, 8.7)	−1.9 (−8.8, 5.0)	−2.6 (−10.3, 5.2)	11.5 (−10.7, 33.6)	1.4 (−4.6, 7.4)	0.5 (−4.9, 6.0)	−0.5 (−5.1, 4.0)
Baseline Activities AFEQT Score												
Model 1 = Social determinant	−8.0 (−18.3, 2.3)	−4.3 (−13.7, 5.0)	−4.1 (−12.0, 3.9)	0.6 (−6.3, 7.4)	−3.1 (−9.1, 2.9)	2.5 (−3.0, 7.9)	−2.3 (−11.5, 6.8)	−0.3 (−10.5, 9.9)	3.3 (−26.5, 33.0)	1.9 (−5.9, 9.6)	0.9 (−6.0, 7.9)	−2.6 (−8.5, 3.3)
Model 2 = Model 1 + age + BMI[e]	−7.2 (−17.6, 3.1)	−3.2 (−12.5, 6.2)	−3.0 (−11.0, 5.0)	1.0 (−5.9, 7.8)	−2.4 (−8.4, 3.6)	2.7 (−2.7, 8.2)	−3.6 (−12.8, 5.7)	−1.9 (−12.3, 8.4)	4.6 (−25.1, 34.3)	1.2 (−6.6, 9.1)	−0.5 (−7.6, 6.7)	−1.8 (−7.8, 4.2)
Model 3 = Model 2 + cardiovascular risk factors[f]	−8.5 (−18.8, 1.9)	−1.1 (−10.5, 8.2)	−1.6 (−9.5, 6.4)	1.3 (−5.6, 8.3)	−1.8 (−7.8, 4.3)	1.5 (−3.9, 7.0)	−3.2 (−12.3, 6.0)	−1.9 (−12.2, 8.4)	9.3 (−20.2, 38.7)	1.3 (−6.7, 9.2)	0.8 (−6.4, 8.1)	−2.1 (−8.1, 3.9)
Model 4 = Model 3 + major cardiovascular adverse events[g]	−8.6 (−18.7, 1.5)	0.1 (−9.0, 9.2)	−0.8 (−8.6, 7.0)	1.6 (−5.2, 8.4)	−1.3 (−7.2, 4.7)	2.3 (−3.2, 7.7)	−2.2 (−11.2, 6.7)	−1.2 (−11.4, 8.9)	4.9 (−24.0, 33.8)	1.2 (−6.6, 9.0)	1.8 (−5.3, 8.9)	−0.7 (−6.7, 5.3)
Baseline Symptoms AFEQT Score												
Model 1 = Social determinant	0.0 (−9.2, 9.3)	−5.0 (−13.4, 3.3)	1.5 (−5.7, 8.6)	−2.2 (−8.3, 3.9)	−2.6 (−7.9, 2.8)	6.7 (1.9, 11.5)	−2.7 (−10.8, 5.4)	−2.5 (−11.6, 6.5)	6.7 (−19.6, 32.9)	3.5 (−3.4, 10.3)	−0.9 (−7.1, 5.4)	−2.6 (−7.9, 2.6)
Model 2 = Model 1 + age + BMI	0.6 (−8.7, 9.8)	−4.2 (−12.5, 4.2)	1.7 (−5.5, 8.8)	−2.3 (−8.4, 3.8)	−2.2 (−7.5, 3.2)	7.0 (2.2, 11.8)	−4.8 (−13.0, 3.3)	−5.0 (−14.2, 4.2)	6.6 (−19.5, 32.7)	1.9 (−5.0, 8.8)	−2.2 (−8.5, 4.1)	−1.1 (−6.5, 4.2)

Model 3 = Model 2 + cardiovascular risk factors	0.0 (−9.2, 9.3)	−3.4 (−11.7, 5.0)	1.8 (−5.4, 8.9)	−2.1 (−8.2, 4.1)	−2.1 (−7.5, 3.3)	6.1 (1.3, 11.0)	−5.3 (−13.4, 2.9)	−4.4 (−13.6, 4.8)	10.6 (−15.4, 36.6)	2.0 (−5.1, 9.0)	−0.8 (−7.2, 5.6)	−1.5 (−6.8, 3.8)
Model 4 = Model 3 + major cardiovascular adverse events	0.3 (−8.9, 9.5)	−2.5 (−10.9, 5.9)	2.2 (−5.0, 9.3)	−2.4 (−8.5, 3.8)	−2.3 (−7.7, 3.1)	7.1 (2.2, 12.0)	−5.3 (−13.4, 2.9)	−4.6 (−13.8, 4.6)	8.2 (−17.8, 34.2)	1.8 (−5.3, 8.8)	−1.0 (−7.4, 5.5)	−1.6 (−7.0, 3.8)
Baseline Treatment AFEQT Score												
Model 1 = Social determinant	−2.8 (−12.0, 6.4)	0.6 (−7.7, 8.9)	1.1 (−6.0, 8.2)	1.5 (−4.6, 7.6)	−3.6 (−8.9, 1.7)	3.5 (−1.3, 8.3)	3.5 (−4.6, 11.5)	0.1 (−8.9, 9.1)	25.3 (−0.9, 51.4)	6.1 (−0.7, 12.9)	1.8 (−4.4, 7.9)	−3.0 (−8.3, 2.2)
Model 2 = Model 1 + age + BMI	−1.1 (−10.2, 7.9)	2.8 (−5.4, 11.0)	2.5 (−4.5, 9.5)	1.7 (−4.2, 7.7)	−2.5 (−7.7, 2.8)	4.0 (−0.7, 8.7)	0.1 (−7.9, 8.1)	−3.9 (−12.8, 5.1)	26.5 (0.8, 52.1)	3.6 (−3.2, 10.4)	−1.2 (−7.4, 5.0)	−0.4 (−5.6, 4.9)
Model 3 = Model 2 + cardiovascular risk factors	−2.5 (−11.8, 6.8)	3.3 (−5.0, 11.6)	2.5 (−4.6, 9.6)	1.7 (−4.5, 7.8)	−2.4 (−7.8, 2.9)	4.2 (−0.7, 9.0)	−0.2 (−8.3, 7.9)	−4.0 (−13.1, 5.1)	27.6 (1.7, 53.5)	3.1 (−3.9, 10.1)	−0.3 (−6.7, 6.1)	−0.4 (−5.7, 5.0)
Model 4 = Model 3 + major cardiovascular adverse events	−2.7 (−12.0, 6.5)	3.6 (−4.7, 11.9)	2.5 (−4.6, 9.6)	1.7 (−4.5, 7.8)	−2.5 (−7.9, 2.9)	4.8 (−0.1, 9.7)	0.3 (−7.8, 8.4)	−3.7 (−12.9, 5.4)	25.7 (−0.2, 51.7)	3.1 (−4.0, 10.1)	−0.1 (−6.5, 6.3)	0.3 (−5.1, 5.7)

Abbreviations: AF = atrial fibrillation, AFEQT = atrial fibrillation effect on quality-of-life, BMI = body mass index, calculated as weight in kilograms divided by height in meters squared, CABG = coronary artery bypass grafting, CI = confidence interval, CKD = chronic kidney disease, COPD = chronic obstructive pulmonary disease, CVA = cerebrovascular event or stroke, HTN = hypertension, NDI = neighborhood deprivation index, PCI = percutaneous coronary intervention, SDoH = social determinants of health, TIA = transient ischemic attack

[a] The NDI quartiles were determined based on comparison of the NDI at the selected census track compared to the national average. The referent group was the bottom quartile of NDI, described as tracks with the least deprivation (n = 69). The below average (n = 63), above average (n = 96), and most deprivation (n = 288) quartiles were considered as ordinal categories for comparison.

[b] These categories were determined by self-report. Non-Hispanic Black (n = 207) and Hispanic/Latinx (n = 125) were compared with Non-Hispanic White (n = 184) participants as the reference for all models.

[c] Public insurance (n = 353) and self-pay (n = 115) insurance were compared against private (n = 48) insurance, which was the reference group for all models.

[d] Language was confirmed via patient interview. Spanish (n = 67) was selected if a translator was required for the interview, or was categorized as 'Other primary langauge' (n = 4). The referent language for models was English (n= 445).

[e] Sociodemographic factors included age, race & ethnicity, and sex, when not included as the primary exposure).

[f] Cardiovascular risk factors included obesity, smoking, hypertension, diabetes, kidney disease, and hyperlipidemia.

[g] The prevalent major cardiovascular adverse events included coronary artery disease, cerebrovascular accident (stroke or TIA), congestive heart failure, or peripheral vascular disease.

Table S10: ***Treatment Strategy over Follow-up Period***

	Overall (n = 516)[a]	Rate-control strategy (n = 328)[a]	Rhythm-control strategy (n = 188)[a]
Sociodemographics			
Age (years)	64 (58, 71)	64 (59, 73)	64 (57, 69)
Sex			
Female	185 (36%)	119 (36%)	66 (35%)
Male	331 (64%)	209 (64%)	122 (65%)
Race & Ethnicity			
Non-Hispanic White	184 (36%)	111 (34%)	73 (39%)
Hispanic/Latinx	125 (24%)	79 (24%)	46 (24%)
Non-Hispanic Black	207 (40%)	138 (42%)	69 (37%)
Neighborhood deprivation index by quartile			
Least deprivation	69 (13%)	40 (12%)	29 (15%)
Below average deprivation	63 (12%)	42 (13%)	21 (11%)
Above average deprivation	96 (19%)	54 (16%)	42 (22%)
Most deprivation	288 (56%)	192 (59%)	96 (51%)
Insurance Class			
Private	48 (9.3%)	28 (8.5%)	20 (11%)
Public	353 (68%)	223 (68%)	130 (69%)
Self-Pay	115 (22%)	77 (23%)	38 (20%)
Language Group			
English	445 (86%)	283 (86%)	162 (86%)
Other primary language	4 (0.8%)	2 (0.6%)	2 (1.1%)
Spanish	67 (13%)	43 (13%)	24 (13%)
Marital Status			
Married or cohabitating	216 (42%)	132 (40%)	84 (45%)
Divorced, separated, or widowed	109 (21%)	78 (24%)	31 (16%)
Single or never-married	191 (37%)	118 (36%)	73 (39%)
Clinical Covariates			
Coronary artery disease	102 (20%)	58 (18%)	44 (24%)
Congestive heart failure	212 (42%)	121 (38%)	91 (49%)
Stroke/TIA	90 (18%)	63 (20%)	27 (14%)
Diabetes mellitus	221 (44%)	138 (43%)	83 (44%)
Hypertension	392 (76%)	242 (74%)	150 (80%)
Chronic kidney disease	139 (28%)	95 (30%)	44 (24%)
COPD	126 (25%)	79 (25%)	47 (25%)
CHA2DS2VASc score	3 (2, 5)	3 (2, 5)	3 (2, 4)
Type of AF			
Paroxysmal	411 (80%)	263 (80%)	148 (79%)
Persistent	105 (20%)	65 (20%)	40 (21%)
Follow-up Medications			
Antiplatelet agents	212 (41%)	147 (45%)	65 (35%)
Anticoagulant agents	353 (68%)	229 (70%)	124 (66%)
Sodium channel blockers	71 (16%)	37 (13%)	34 (22%)
Beta blockers	346 (67%)	227 (69%)	119 (63%)
Potassium channel blockers	134 (26%)	58 (18%)	76 (40%)
Calcium channel blockers	56 (11%)	35 (11%)	21 (11%)
Follow-up Treatment Strategy			

Rate-control medications	360 (70%)	234 (71%)	126 (67%)
Rhythm-control medications	179 (40%)	81 (28%)	98 (63%)
Catheter ablation	68 (13%)	33 (10%)	35 (19%)
Electrical direct-current cardioversion	54 (10%)	38 (12%)	16 (8.5%)
Baseline AFEQT Scores			
Overall	79 (57, 92)	80 (59, 93)	75 (53, 92)
Symptoms	88 (67, 100)	88 (67, 100)	88 (62, 100)
Daily activities	73 (44, 96)	74 (48, 96)	73 (39, 94)
Treatment concerns	83 (61, 100)	86 (64, 100)	81 (56, 100)
Changes in AFEQT Scores			
Overall (change)	5 (-5, 20)	4 (-4, 19)	5 (-7, 21)
Symptoms (change)	0 (-4, 17)	0 (-2, 17)	0 (-4, 21)
Daily activities (change)	2 (-8, 25)	0 (-10, 23)	7 (-8, 30)
Treatment concerns (change)	0 (-6, 22)	3 (-3, 22)	0 (-8, 22)

Abbreviations: AF = atrial fibrillation, AFEQT = atrial fibrillation effect on quality-of-life, BMI = body mass index, calculated as weight in kilograms divided by height in meters squared, CABG = coronary artery bypass grafting, CI = confidence interval, CKD = chronic kidney disease, COPD = chronic obstructive pulmonary disease, CVA = cerebrovascular event or stroke, HTN = hypertension, NDI = neighborhood deprivation index, PCI = percutaneous coronary intervention, SDoH = social determinants of health, TIA = transient ischemic attack

[a] Median (Q1, Q3); n (%)

Table S11: ***Rhythm-Control Strategy and Social Determinants of Health***

	NDI Quartile (Least deprivation)[a]			Race & Ethnicity (Non-Hispanic White)[b]		Sex (Female)	Insurance (Private)[c]		Language (English)[d]		Marital Status (Married or cohabitating)	
	Below average deprivation	Above average deprivation	Most deprivation	Hispanic/Latinx	Non-Hispanic Black	Male	Public	Self-Pay	Other primary language	Spanish	Divorced, separated, or widowed	Single or never-married
Baseline Rhythm-Control Strategy[e]												
Model 1 = Adjustment for baseline AFEQT[f]	0.7 (0.3, 1.4)	1.0 (0.6, 2.0)	0.7 (0.4, 1.2)	0.9 (0.6, 1.4)	0.7 (0.5, 1.1)	1.1 (0.7, 1.6)	0.8 (0.4, 1.5)	0.7 (0.3, 1.4)	1.9 (0.2, 15.6)	1.0 (0.6, 1.7)	0.6 (0.4, 1.0)	1.0 (0.6, 1.4)
Model 2 = Model 1 + age + BMI + sociodemographic factors[g]	0.6 (0.3, 1.3)	1.0 (0.5, 1.8)	0.6 (0.4, 1.1)	0.9 (0.5, 1.4)	0.7 (0.5, 1.1)	1.1 (0.7, 1.5)	0.9 (0.5, 1.8)	0.8 (0.4, 1.6)	1.8 (0.2, 14.9)	1.1 (0.6, 1.9)	0.7 (0.4, 1.1)	0.9 (0.6, 1.3)
Model 3 = Model 2 + cardiovascular risk factors[h]	0.7 (0.3, 1.4)	0.9 (0.5, 1.8)	0.6 (0.3, 1.1)	0.9 (0.6, 1.5)	0.7 (0.5, 1.1)	1.0 (0.7, 1.5)	1.0 (0.5, 1.8)	0.9 (0.4, 1.9)	1.5 (0.2, 13.0)	1.1 (0.6, 1.9)	0.8 (0.5, 1.3)	0.9 (0.6, 1.3)
Model 4 = Model 3 + major cardiovascular adverse events[i]	0.7 (0.3, 1.4)	0.9 (0.5, 1.7)	0.6 (0.3, 1.0)	0.9 (0.6, 1.5)	0.7 (0.4, 1.1)	1.0 (0.7, 1.4)	0.9 (0.5, 1.8)	0.9 (0.4, 1.8)	1.9 (0.2, 17.2)	1.1 (0.6, 1.9)	0.7 (0.4, 1.3)	0.8 (0.5, 1.3)
Follow-up Rhythm-Control Strategy[e]												
Model 1 = Adjustment for baseline AFEQT[f]	1.5 (0.7, 3.1)	1.1 (0.6, 2.1)	1.0 (0.6, 1.8)	0.7 (0.4, 1.1)	0.6 (0.4, 0.9)	1.3 (0.9, 2.0)	1.3 (0.7, 2.5)	0.8 (0.4, 1.5)	2.6 (0.3, 53.5)	0.7 (0.4, 1.1)	0.6 (0.4, 0.9)	0.9 (0.6, 1.4)
Model 2 = Model 1 + age + BMI + sociodemographic factors[g]	1.4 (0.7, 3.0)	1.0 (0.5, 2.0)	1.0 (0.6, 1.7)	0.7 (0.4, 1.1)	0.6 (0.4, 0.9)	1.3 (0.9, 1.9)	1.4 (0.7, 2.7)	0.8 (0.4, 1.7)	2.6 (0.3, 52.4)	0.7 (0.4, 1.2)	0.6 (0.4, 1.0)	0.9 (0.6, 1.4)
Model 3 = Model 2 + cardiovascular risk factors[h]	1.7 (0.8, 3.6)	1.0 (0.5, 1.9)	1.1 (0.6, 1.9)	0.7 (0.4, 1.2)	0.6 (0.4, 0.9)	1.3 (0.9, 1.9)	1.5 (0.8, 2.8)	0.8 (0.4, 1.7)	2.1 (0.3, 44.2)	0.6 (0.3, 1.1)	0.7 (0.4, 1.1)	0.9 (0.6, 1.4)
Model 4 = Model 3 + major cardiovascular adverse events[i]	1.7 (0.8, 3.8)	0.9 (0.5, 1.9)	1.0 (0.6, 1.8)	0.7 (0.4, 1.1)	0.6 (0.4, 0.9)	1.3 (0.9, 2.0)	1.5 (0.8, 2.9)	0.8 (0.4, 1.7)	2.7 (0.3, 56.1)	0.6 (0.3, 1.0)	0.6 (0.4, 1.1)	0.9 (0.6, 1.4)

Abbreviations: AF = atrial fibrillation, AFEQT = atrial fibrillation effect on quality-of-life, BMI = body mass index, calculated as weight in kilograms divided by height in meters squared, CABG = coronary artery bypass grafting, CI = confidence interval, CKD = chronic kidney disease, COPD = chronic obstructive pulmonary disease, CVA = cerebrovascular event or stroke, HTN = hypertension, NDI = neighborhood deprivation index, PCI = percutaneous coronary intervention, SDoH = social determinants of health, TIA = transient ischemic attack

[a] The NDI quartiles were determined based on comparison of the NDI at the selected census track compared to the national average. The referent group was the bottom quartile of NDI, described as tracks with the least deprivation (n = 69). The below average (n = 63), above average (n = 96), and most deprivation (n = 288) quartiles were considered as ordinal categories for comparison.

[b] These categories were determined by self-report. Non-Hispanic Black (n = 207) and Hispanic/Latinx (n = 125) were compared with Non-Hispanic White (n = 184) participants as the reference for all models.

[c] Public insurance (n = 353) and self-pay (n = 115) insurance were compared against private (n = 48) insurance, which was the reference group for all models.

[d] Language was confirmed via patient interview. Spanish (n = 67) was selected if a translator was required for the interview, or was categorized as 'Other primary langauge' (n = 4). The referent language for models was English (n= 445).

[e] Generalized mixed effects models for this binary outcome were used, with random effects for site and subtype of AF. Odds ratios (95% CI) are presented.

[f] As the outcome was defined as change in AFEQT over study period, all models adjusted for baseline AFEQT scores.

[g] Sociodemographic factors included age, race & ethnicity, and sex, when not included as the primary exposure).

[h] Cardiovascular risk factors included obesity, smoking, hypertension, diabetes, kidney disease, and hyperlipidemia.

[i] The prevalent major cardiovascular adverse events included coronary artery disease, cerebrovascular accident (stroke or TIA), congestive heart failure, or peripheral vascular disease.

Statistical Software

```
R version 4.5.1 (2025-06-13)
Platform: x86_64-apple-darwin20
Running under: macOS Sequoia 15.4

Matrix products: default
BLAS:   /Library/Frameworks/R.framework/Versions/4.5-x86_64/Resources/lib/libRblas.0.dyli
b
LAPACK: /Library/Frameworks/R.framework/Versions/4.5-x86_64/Resources/lib/libRlapack.dyli
b;  LAPACK version 3.12.1

locale:
[1] en_US.UTF-8/en_US.UTF-8/en_US.UTF-8/C/en_US.UTF-8/en_US.UTF-8

time zone: America/Denver
tzcode source: internal

attached base packages:
[1] stats     graphics  grDevices utils     datasets  methods   base

other attached packages:
 [1] rmdl_0.1.0.9000    vctrs_0.6.5        xfun_0.52          consort_1.2.2
 [5] gtsummary_2.3.0    gt_1.0.0           tarchetypes_0.13.1 targets_1.11.3
 [9] glue_1.8.0         patchwork_1.3.1    yardstick_1.3.2    workflowsets_1.1.1
[13] workflows_1.2.0    tune_1.3.0         rsample_1.3.0      recipes_1.3.1
[17] parsnip_1.3.2      modeldata_1.4.0    infer_1.0.9        dials_1.4.0
[21] scales_1.4.0       broom_1.0.8        tidymodels_1.3.0   lubridate_1.9.4
[25] forcats_1.0.0      stringr_1.5.1      dplyr_1.1.4        purrr_1.1.0
[29] readr_2.1.5        tidyr_1.3.1        tibble_3.3.0       ggplot2_3.5.2
[33] tidyverse_2.0.0

loaded via a namespace (and not attached):
 [1] rlang_1.1.6         magrittr_2.0.3      furrr_0.3.1
 [4] compiler_4.5.1      mgcv_1.9-3          callr_3.7.6
 [7] lhs_1.2.0           pkgconfig_2.0.3     fastmap_1.2.0
[10] backports_1.5.0     labeling_0.4.3      rmarkdown_2.29
[13] markdown_2.0        prodlim_2025.04.28  tzdb_0.5.0
[16] ps_1.9.1            litedown_0.7        jsonlite_2.0.0
[19] parallel_4.5.1      prettyunits_1.2.0   R6_2.6.1
[22] stringi_1.8.7       RColorBrewer_1.1-3  parallelly_1.45.1
[25] rpart_4.1.24        Rcpp_1.1.0          iterators_1.0.14
[28] knitr_1.50          future.apply_1.20.0 base64enc_0.1-3
[31] Matrix_1.7-3        splines_4.5.1       nnet_7.3-20
[34] igraph_2.1.4        timechange_0.3.0    tidyselect_1.2.1
[37] rstudioapi_0.17.1   dichromat_2.0-0.1   yaml_2.3.10
[40] timeDate_4041.110   codetools_0.2-20    curl_6.4.0
[43] processx_3.8.6      listenv_0.9.1       lattice_0.22-7
[46] withr_3.0.2         evaluate_1.0.4      future_1.58.0
[49] survival_3.8-3      xml2_1.3.8          pillar_1.11.0
[52] foreach_1.5.2       insight_1.3.1       generics_0.1.4
[55] rprojroot_2.1.0     hms_1.1.3           commonmark_2.0.0
[58] juicyjuice_0.1.0    globals_0.18.0      base64url_1.4
[61] class_7.3-23        tools_4.5.1         see_0.11.0
[64] data.table_1.17.8   gower_1.0.2         fs_1.6.6
```

```
[67] grid_4.5.1          cards_0.6.1         ipred_0.9-15
[70] nlme_3.1-168        cli_3.6.5           DiceDesign_1.10
[73] lava_1.8.1          V8_6.0.4            gtable_0.3.6
[76] GPfit_1.0-9         sass_0.4.10         digest_0.6.37
[79] farver_2.1.2        htmltools_0.5.8.1   lifecycle_1.0.4
[82] here_1.0.1          hardhat_1.4.1       secretbase_1.0.5
[85] MASS_7.3-65
```
